# 3D Printing Technologies for Fabrication of Magnetic Materials Based on Metal–Polymer Composites: A Review

**DOI:** 10.3390/ma16216928

**Published:** 2023-10-28

**Authors:** Alina Mazeeva, Dmitriy Masaylo, Nikolay Razumov, Gleb Konov, Anatoliy Popovich

**Affiliations:** Institute of Machinery, Materials and Transport, Peter the Great St. Petersburg Polytechnic University, 29, Polytechnicheskaya Str., 195251 Saint Petersburg, Russia; dmasaylo@gmail.com (D.M.); n.razumov@inbox.ru (N.R.); 071133gleb@mail.ru (G.K.); popovicha@mail.ru (A.P.)

**Keywords:** 3D printing, additive manufacturing, fused deposition modeling, extrusion, magnetic materials, polymer–metal magnetic composites, polymer-based magnetic composites, powder

## Abstract

Additive manufacturing is a very rapidly developing industrial field. It opens many possibilities for the fast fabrication of complex-shaped products and devices, including functional materials and smart structures. This paper presents an overview of polymer 3D printing technologies currently used to produce magnetic materials and devices based on them. Technologies such as filament-fused modeling (FDM), direct ink writing (DIW), stereolithography (SLA), and binder jetting (BJ) are discussed. Their technological features, such as the optimal concentration of the filler, the shape and size of the filler particles, printing modes, etc., are considered to obtain bulk products with a high degree of detail and with a high level of magnetic properties. The polymer 3D technologies are compared with conventional technologies for manufacturing polymer-bonded magnets and with metal 3D technologies. This paper shows prospective areas of application of 3D polymer technologies for fabricating the magnetic elements of complex shapes, such as shim elements with an optimized shape and topology; advanced transformer cores; sensors; and, in particular, the fabrication of soft robots with a fast response to magnetic stimuli and composites based on smart fillers.

## 1. Introduction

Today, additive technologies (ATs) are among the most actively developing fields of mechanical engineering and metallurgy. At this stage, a large number of studies make it possible to fabricate critical parts for the power engineering, shipbuilding, aerospace, medicine, and other industries [1,2,3,4]. One of the main advantages of ATs is the ability to manufacture parts of almost any shape without preliminarily manufacturing special equipment. In this way, it is possible to produce unique parts with complex shapes with minimal waste of materials and minimal time consumption, which brings progress in mechanical engineering to a new level. 

Magnetic materials are usually considered as functional ones and rarely have special structural designs. However, with the development of ATs and with the active study of their possibilities in material engineering, a whole group of studies have appeared, devoted to the application of ATs to the manufacture of electrical parts from various electrical and magnetic materials, in particular, rotors of complex shapes [5,6,7,8,9,10]. In addition, ATs offer the possibility of the fairly quick and relatively low-cost manufacturing of complex structures such as lattice structures with different types of cells. This makes it possible to reduce the total weight of electric motors that results in a reduction in its inertia, while maintaining a sufficient level of mechanical properties [11,12]. A special field of AT application is the manufacture of various soft robots, where magnetic materials can be used.

Studies also show that, in addition to the fabrication of complex structures, ATs can be used to manipulate materials’ properties, especially with respect to metal alloys [13,14,15]. In particular, many magnetic characteristics are very sensitive to the structural state. They can change significantly not only when one AT is replaced by another one but also when they depend on the technological parameters of a certain AT. This means that material science aspects are very important when using ATs for fabricating products from metallic materials, especially magnetic ones. There are also studies aimed at tailoring known alloy compositions for use in additive technologies [16].

Most papers on additive manufacturing (AM) of magnetic materials are devoted to hard magnetic materials based on an Nd-Fe-B system [17,18,19] and soft magnetic materials based on permalloys [14,20,21,22,23], since they currently have the best magnetic characteristics in their groups. However, researchers are also searching for other magnetic materials and their use possibilities for AM. In particular, their interest is directed to rare-earth-free hard magnetic materials from the well-known Alnico alloys [24,25] to exotic Mn-based magnets [26,27,28,29,30]. In the field of soft magnetic materials, the AM of amorphous alloys [31,32,33] and smart materials [15,34,35,36,37] is garnering more attention.

Nowadays, there are more than 50 types of 3D printing technologies based on various principles, which can be divided into the following main groups: material inkjet spraying (MJ), material extrusion (ME), vat photopolymerization, binder jetting (BJ), powder bed fusion (PBF), directed energy deposition (DED), and layer-by-layer lamination [38]. From these methods, several more specialized technologies have emerged that are widely used at the moment. They are fused deposition modeling (FDM), direct ink writing (DIW), stereolithography (SLA), two-photon polymerization (TPP), inkjet printing (IJP), selective laser sintering/melting (SLS/SLM), and so on [39]. The most commonly used technologies for producing products from metallic materials are DED and PBF technologies with a laser or an electron beam as an energy source. These technologies can be called “metal” ATs and are used for fabricating metallic magnetic materials as well. In particular, the production of permanent magnets based on Nd-Fe-B by these methods makes it possible to obtain properties close to those of sintered magnets manufactured by conventional methods of powder metallurgy. However, ATs that include the use of some kind of polymeric binder, such as FDM, DIW, SLA, and BJ, are usually more affordable both in terms of equipment and the main and consumable materials used. Despite the fact that magnets fabricated by any of these technologies have lower properties than sintered magnets and magnets fabricated by metal ATs, polymer ATs make it possible to quickly and cost-effectively produce a great variety of complex structures [40], and they are indispensable for the manufacture of soft robots with a fast response due to the action of an external magnetic field.

This work aims to review the current state of the art in the use of various polymer ATs for the manufacturing of magnetic materials and parts, their features, and their possible use in the future. It includes some of the newest papers in this field and is mainly focused on the technological aspects of various techniques and their influence on magnetic characteristics to make it helpful in the real industry of magnetic materials and smart structures. 

## 2. Magnetic Parts of Complex Shapes

Magnetic materials are often functional ones and usually have a rather simple or standard shape. They can be used in the form of sheets; plates; bars; or coatings, including composite ones. However, the complex geometry of a magnet can help to achieve the maximum reduction in stray magnetic fields in certain directions, which is particularly important for sensors based on giant magnetic impedance (GMI) [41,42]. 

To fabricate electro-technical products from magnetic materials, it is critical to develop a preliminary simulation-based design [41,43,44,45,46,47,48]. This allows us to determine the optimal shape of the parts providing specific external magnetic characteristics of the product.

In [41,48], the shape and topology of a permanent magnet based on Nd-Fe-B were optimized using computer modeling. The main task of developing an optimal magnet shape is to ensure minimal stray fields. For modeling, a special software module was developed, which was based on classical equations [41,48]:

(1) $$\vec{h}=-\nabla u,$$

where $\vec{h}$ is the stray field and *u* is the scalar magnetic potential, defined by Poisson’s ratio:

(2) $$\Delta u=\nabla \cdot \vec{M},$$

where $\vec{M}$ is the magnetization, with the following boundary conditions:

(3) $$u(\vec{x})=O(\frac{1}{\left|\vec{x}\right|}),\;\vec{x}\;\to \;\infty$$

**Figure 1:**
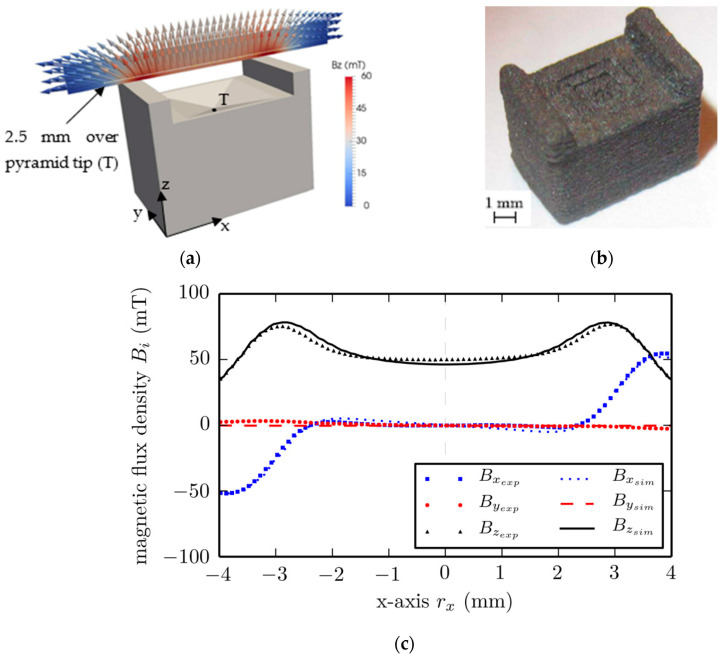
(**a**) Developed shape of a parallelepiped magnet with a pyramidal recess; (**b**) FDM-printed isotropic magnet with filament containing spherical NdFeB particles in a PA11 matrix; (**c**) simulation and experimental data comparison for B_x_, B_y_, and B_z_ components along *x* axis of the magnet [41].

To solve the Maxwell magnetostatic equations, the finite elements method (FEM) is widely applied. There are several approved algorithms for stray field simulation described in [49]. In [41], the researchers used a simulation to determine the optimal shape for permanent magnets and found that the magnet magnetized in an external magnetic field 4T along the *z* axis should be a parallelepiped with a pyramidal recess (Figure 1a). FDM technology allowed the magnet to be fabricated according to the designed model with a rather good precision (Figure 1b). The simulated and experimental values of magnetic flux density B along the magnet’s length at the height of 2.5 mm above the recess tip T coincided rather well (Figure 1c). This indicates *the feasibility of a combined approach of using the preliminary design of a magnet’s complex geometry and subsequent polymer 3D printing with high accuracy to obtain a given profile of magnetic flux distribution in an external space even in the case of miniature products.* The overall dimensions of the printed magnet in [41] were 7 × 5 × 5.5 mm, with a detail grade of 0.8 mm.

The magnetic field generated by permanent magnets is an important component in various methods and experiments of magnetic analysis in fields such as nuclear magnetic resonance (NMR), magnetic resonance imaging (MRI), magnetometers, neutron interferometers, magnetic traps, particle counters, etc. For most of these applications, the homogeneity of the magnetic field is rather critical [45]. The technique for correcting field inhomogeneity is commonly referred to as magnetic system shimming. There are two main shimming methods to improve the homogeneity of the magnetic field of a permanent magnet:

(1) Passive shimming corrects the magnetic field by using ferromagnetic materials placed at specific locations along the magnet [50,51].
(2) Active shimming uses electromagnets with special coils to create a correcting magnetic field [50,52,53].

For passive shimming, it is necessary to have a magnet of a certain complex shape, which forms the required external magnetization distribution. To search for such a shape, various numerical and computer simulation methods are used. They include algorithms for optimizing the magnetization created following a certain design and for optimizing an existing structure, the location of the magnets, and its topology, *which makes it possible to create a complex shape for a magnet from scratch* [50,54,55].

**Figure 2:**
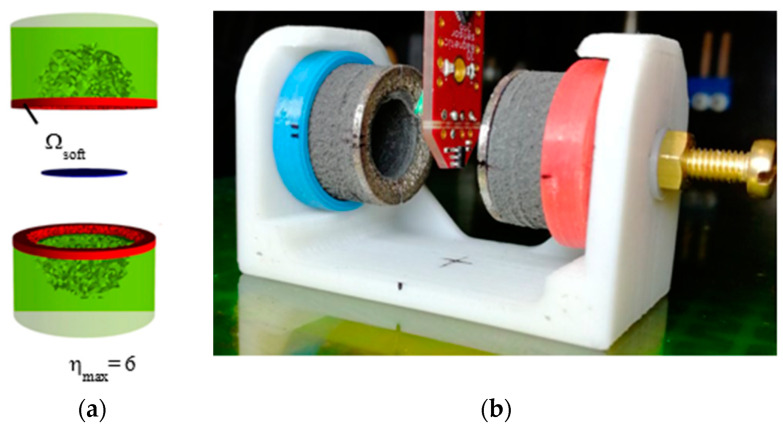
(**a**) Simulated magnet topology with shimming element; (**b**) printed magnet system with the optimized topology [45].

In [50], the topology of the initial cylindrical magnets was optimized by a computer simulation of the inverse problem of stray fields. As a result of topological optimization, a CAD model of shimming elements made of soft magnetic material was designed, and then, the elements were printed and attached to permanent magnets (Figure 2).

The printing of a soft magnetic shimming element using the FDM method with a metal–polymer composite in [50] resulted in a too-high coercive force and a too-low magnetic permeability, which led the authors to the idea of using the SLM method to print this part. When measuring the uniformity of the magnetic field between two magnets with shimming elements, the maximum inhomogeneity *η* was 6 when determining the inhomogeneity with the following equation:

(4) $$\eta =\frac{B_{max}-B_{min}}{B_{mean}},$$

Before shimming, this value was 210 [50].

In [55], the density method known as solid isotropic microstructure with penalization (SIMP) was used to optimize the topology. This method takes into account the material density in each element, which ranges from 0 (void) to 1 (bulk). This results in one optimization per element. Intermediate densities are neglected in this approach, which means that the density of the final model should only be 0 or 1. For the simulation of a permanent magnet, the magnetization of one element Ω_m_ with the density *ρ* = 0;1 is determined with the following equation:

(5) $$\vec{M}\left(\rho \right)=\rho^{p}{\vec{M}}_{0},$$

where *p* = 1 is the penalization parameter.

The topological optimization problem used to find the minimum of objective function *J* is

(6) $${min}_{\rho }J(\rho ),$$

at the following condition:

(7) $$\int_{\Omega_{m}}\rho \left(\vec{r}\right)d\vec{r}\;\leq \;V$$

$$0\;\leq \;(\vec{r})\;\leq \;1,\;\vec{r}\;\in \;\Omega_{m}$$

where V is the total volume of the magnet.

**Figure 3:**
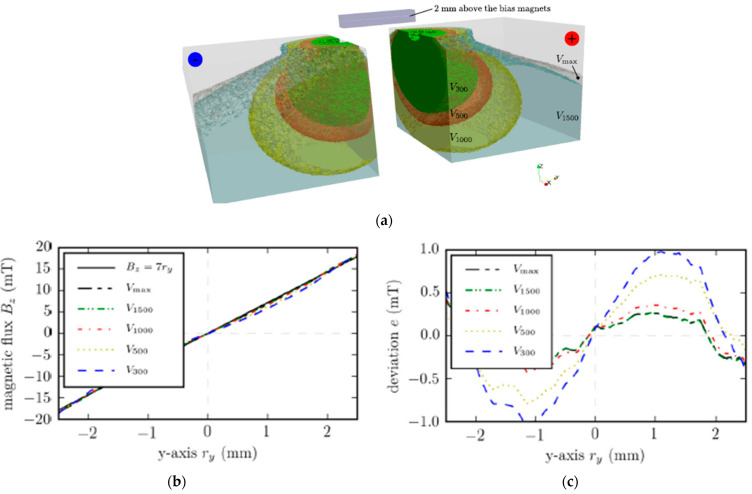
Topology-optimized magnetic configuration with different volume constraints. (**a**) Picture of the topology with different volume constraints. (**b**) Stray field Bz 2 mm above the magnets with different volume constraints. (**c**) Deviation between simulation and the given linearly increasing field [55].

To solve this problem, FEM algorithms are also used. In [55], a special framework was developed to imply this method. Figure 3 shows the simulated model of a magnet with a topology that was optimized to generate a homogeneous linear field in a wide spacial range at minimal total volume.

**Figure 4:**
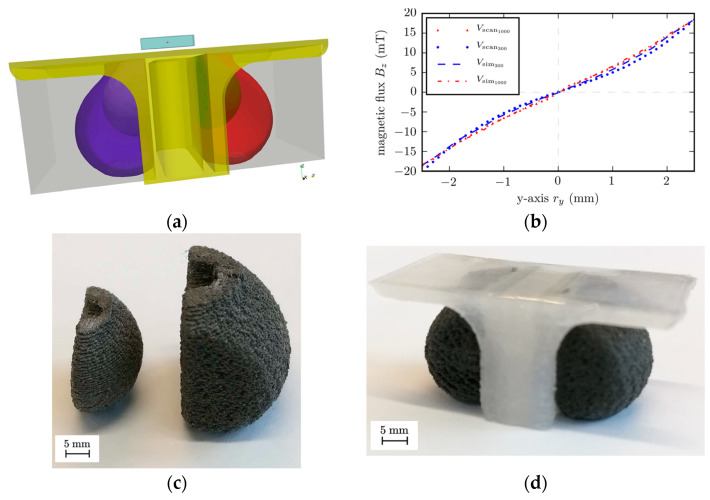
Comparison between simulations and measurements of the printed magnetic setup. (**a**) Setup for two different volume constraints (V1000, V300). (**b**) Line scan of the external field Bz 2 mm above the system compared with simulation results for both volume constraints. (**c**) Picture of the magnets for both constraints (right: V1000, left: V300). (**d**) Picture of the whole magnetic setup for V1000 [55].

Figure 4 shows magnets printed according to the designed CAD model and the assembled magnetic device (Figure 4d). The magnetic flux density values obtained from the simulation and experiments suggest good data convergence and the possibility of using 3D printing to obtain a precisely specified topology. Such a magnetic assembly can be used in field linearity control systems, where high uniformity of magnetic fields is required.

In addition to an external complex shape of the product, *computer modeling makes it possible to design the internal structure of bonded magnets*. The magnetic characteristics of a composite material are determined only by magnetic filler particles, as a polymer matrix is not magnetic. They depend on the volume fraction of magnetic particles and their relative position in the composite structure. To use this features in practice, polymer ATs can be applied to produce gradient structures in one building operation [48].

In [48], a permanent magnet with a variable volume fraction of Nd-Fe-B magnetic particles along the printing direction was designed. *This approach seems promising for forming the required magnetic flux distribution around a permanent magnet without changing its topology.* To determine the optimal gradient of the magnetic particle content in the polymer matrix, a simulation of stray fields was also used. 

The direct problem concludes in the determination of stray fields at a given magnetization and is uniquely solved by FEM [49]. However, the inverse problem of magnetization determination inside a product at a given external magnetic field is nontrivial and can have several solutions. There are several adjoin methods for solving it [49,56,57]; however, to obtain an adequate solution, additional conditions must be specified. For example, in [48], Tikhonov regularization was used:

(8) $$\left(\int_{\Omega_{h}}{\left\|{\vec{h}}_{sim}-{\vec{h}}_{exp}\right\|}^{2}d\vec{r}+\alpha \int_{\Omega_{m}}{\left\|\nabla \vec{M}\right\|}^{2}d\vec{r}\right)$$

where ${\vec{h}}_{sim},\;{\vec{h}}_{exp}$ are the simulated and experimentally measured stray fields, *Ω_m_* is the magnetic body region, *Ω_h_* is the measurement region, and *α* is the optimized Tikhonov regularization parameter.

**Figure 5:**
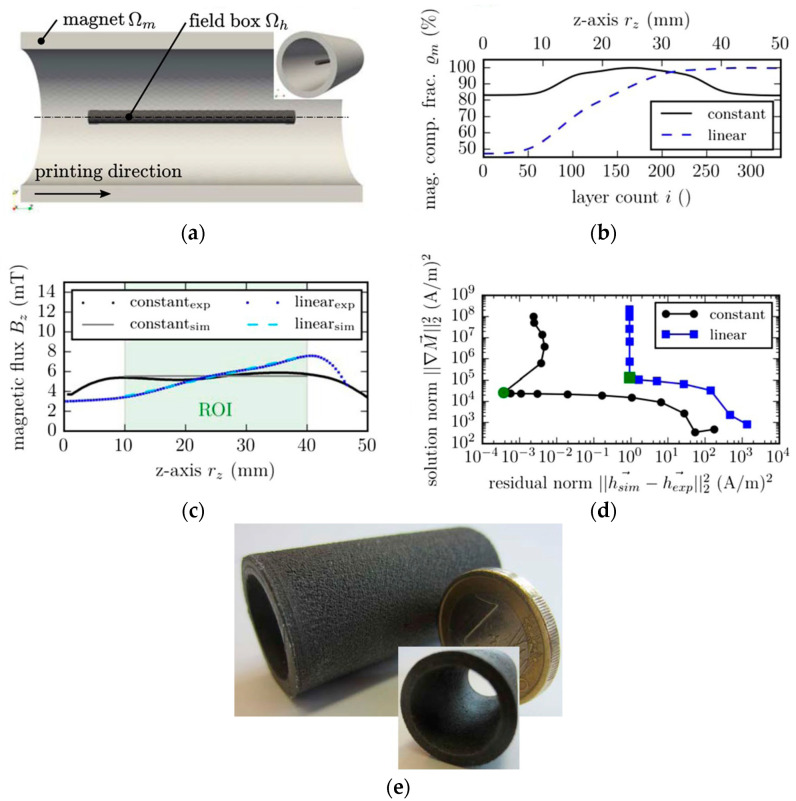
Three-dimensional prints of a magnetic hollow cylinder with a variable magnetic compound fraction distribution to generate a predefined stray field inside the cylinder. (**a**) Model of the hollow cylinder magnet with the dimension in mm (∅25, ∅20, 50 (d_outer_, d_inner_, L)) with a predefined stray field in the field box (∅2, 30 (d, L)). (**b**) Magnetic compound fraction distribution ρ_m_ along the *z*-axis r_z_ to create a constant and linear stray field in the field box. (**c**) Stray field measurements of B_z_ compared with inverse stray field FEM simulations in the middle of the hollow cylinder for the linear and constant field generation magnet. (**d**) L-curve for both designs to find the optimal Tikhonov regularization parameter α. (**e**) Picture of the hollow cylindrical magnet [48].

The inverse problem was solved with a continuum approach that does not take into account particle sizes in a real magnet but takes into account the magnetic composite density distribution generating a correspondent magnetic flux field. Using this method, it is possible not only to simulate the stray fields of a magnet with a known shape but also to design new magnets with a given distribution of stray fields. In [48], a magnet in the form of a hollow cylinder with an optimal magnetization distribution was designed and manufactured (Figure 5).

The authors of [41,48] showed good agreement in their simulation results with their experimental data. Three-dimensional printing technologies make it possible to manufacture magnets according to a developed CAD model with a specified distribution of magnetic particle contents in a polymer matrix by using multimaterial printers [48].

In [58], the use of ATs, in particular, the FDM technology, was studied to improve the Halbach magnetic assembly. The Halbach magnetic assembly is a special configuration of permanent magnets, characterized by the fact that the magnetic field on one of its sides is almost completely absent due to the special arrangement of the assembly elements, and it is homogeneous inside. The Halbach assembly can be used to focus or defocus particles with a spin and to split or merge their beams, as well as to generate alternating magnetic fields for neutron reflectometry [59].

**Figure 6:**
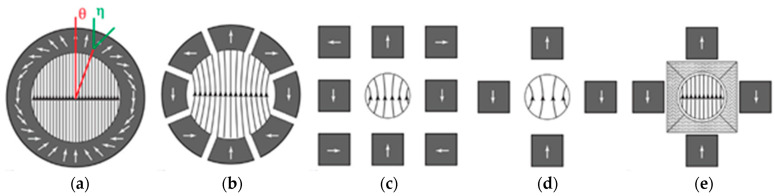
(**a**) Simplification process of a Halbach design and schematic illustration of ideal continuous cylindrical Halbach magnet; (**b**) discretized single cylindrical Halbach magnet in eight pieces; (**c**,**d**) discretized single cylindrical Halbach magnet made with cuboid block magnets; (**e**) effect of shimming the design to increase the uniformity of the magnetic field in an area of interest (dashed area is representative of a shimming structure). The magnetization of each structure is represented by the white arrows. The solid black lines illustrate the dipole magnetic fields obtained within the plane of the Halbach assemblies [58].

The Halbach assembly is usually a long cylinder made of magnetic material with a hole along the axis where a strong magnetic field is present. The magnetic material of the cylinder around the hole is magnetized in such a way that the angle η between the magnetization vector at any point with the angle Θ between the magnetization vector and the vertical line is equal to 2Θ (Figure 6a).

In practice, Halbach assemblies are simpler than the continuous cylinder (Figure 6b–d). The simpler the configuration is and the less magnetic blocks used, the cheaper the manufacturing process is. However, the simpler the configuration is, the less uniform the field becomes at the center of the assembly. As mentioned above, one of the ways to increase the uniformity of the magnetic field is to use shimming inserts with a topology optimized based on simulation. Figure 6e shows an option with a shimming insert made using the FDM method from a metal–polymer composite with a NdFeB filler. This insert tends to improve the uniformity of the magnetic field in a rather simple Halbach assembly, consisting of only four cubic magnetic blocks. The use of the hard magnetic shimming method with the optimal configuration of the shimming element made it possible to increase the average value of magnetic induction by 13% and, more importantly, to increase the magnetic uniformity in the region of interest by 43% [58].

**Figure 7:**
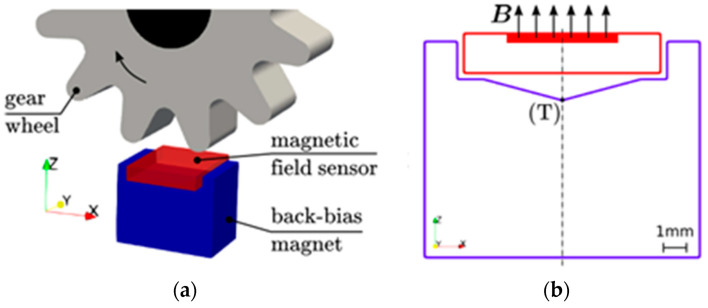
(**a**) Magnetic wheel speed sensing. Principle of the magnetic speed sensing. A permanent magnet is underneath the magnetic field sensor (back-bias magnet). A soft magnetic gear periodically modulates the bias field of the magnet; (**b**) special back-bias magnet design for giant magnetoresistance (GMR) sensors [61].

In [60,61], ATs were used to create the sensor system of a speed wheel (Figure 7). Such high-precision sensor systems are fixed into many devices, especially in cars, for example, in the anti-lock braking system (ABS) or engine control system [42].

A possible design for such speed sensors consists of a magnetic field sensor, such as a Hall sensor or a giant magnetoresistance (GMR) sensor, a permanent magnet that provides a bias field, and a soft magnetic wheel. The magnet is usually located under the sensor (reverse bias magnet) and the rotating soft magnetic wheel changes the magnetic field of the reverse bias magnet. The speed of the wheel rotation is directly proportional to the change in the field. GMR sensors are in-plane sensitive and the linear range is very small [62]. This means that a reverse bias magnet must have very low in-plane magnetic field components. This can be achieved due to the special design of the magnet.

**Figure 8:**
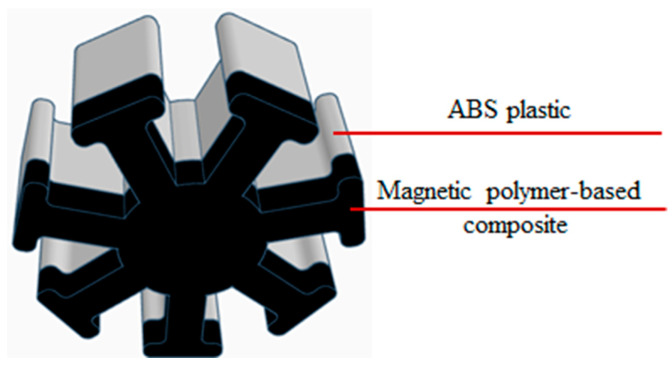
Scheme of the flow sensor propeller with the interface between ABS and magnetic composite according to [65].

The authors of [63] investigated the use of microstereolithography (MSL) for fabricating miniature flow sensors and anisotropic magnetoresistive sensors for detecting rotating magnetic fields. This method allows for obtaining a good degree of detail, but the resulting products are very limited in size, and such products can only be used as miniature sensors in laboratory setups [64]. In [65], the FDM method was used to fabricate a similar flow sensor, but larger in size. In this case, the approach of multimaterial printing was used. The main body of the sensor propeller was printed from acrylonitrile butadiene styrene (ABS), and then, a magnetic composite with a polymer matrix and a magnetic filler was applied to one of the surfaces (Figure 8).

This design allows the sensor to have the mechanical properties of ABS plastic and to give it functionality, which requires a fairly small amount of the magnetic material. The liquid flow through the device results in its rotation, and the magnetic component of the propeller generates alternating magnetic fields, which are detected by the Hall sensor. The frequency of their change is proportional to the liquid flow velocity, which makes it possible to measure this flow.

Polymer–matrix composites are often used to develop advanced scaffolds for bone augmentation [66]. *The FDM and SLA methods make it possible to manufacture products with a given level of porosity*, which should be up to 70% in such products. Magnetic nanoparticles introduced into the polymer matrix allow for controlling the growth of bone tissue using an external magnetic field [66].

Even the manufacture of rather simple structures, for example, transformer cores, is often more expensive if conventional technologies are used rather than ATs [67]. Transformer cores are usually made in stacks in the form of pressed magnetically soft plates assembled in a package. Such cores are open-circuit magnetic systems, which leads to an increase in magnetic energy losses. In general, it is a common problem of transformers, and great efforts have been made to solve it. Theoretically, the ring-shaped closed core allows for obtaining the maximum efficiency of the transformer due to the ability tomaximize the magnetic flux in the coil. However, polymer ATs, for example FDM, make it possible to obtain the required core shape at minimal cost.

Additive technologies can be used not only to manufacture individual parts of complex shapes, which must have a certain set of structural and functional properties, but also to fabricate entire products, such as magnetically driven rotary blood pumps [68] and magnetic soft chain robots (MaSoChains) [69]. Multimaterial 3D printing can also be used for these purposes [69].

Thus, polymer ATs have great advantages in the production of magnetic devices of almost any designed shape, which is often unique and impossible to be fabricated with conventional techniques. In addition, they help to design and materialize the internal structure of the products including gradients in the filler volume fraction and porosity, which help to improve the general performance of fabricated products.

## 3. Fused Deposition Modeling

**Figure 9:**
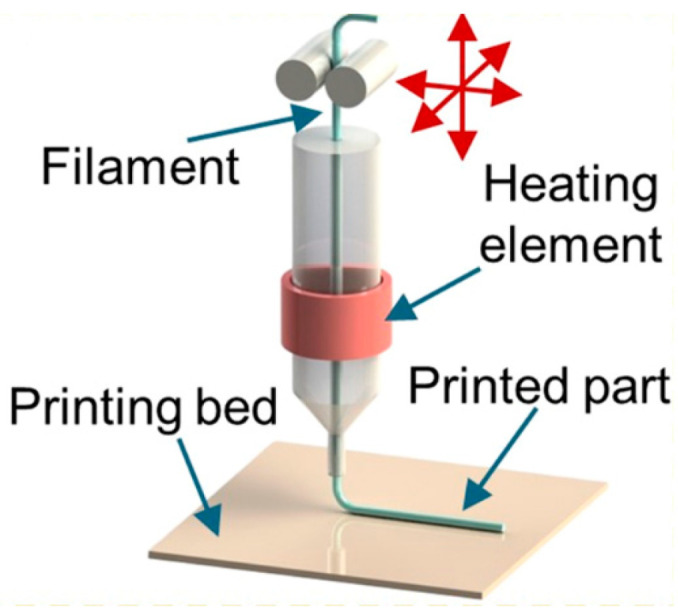
Concept scheme of FDM [70].

FDM technology is one of the most wide-spread and affordable polymer 3D printing technologies; its scheme is shown in Figure 9. It is also often called the fused filament fabricating (FFF) technology. It is based on the use of a thermoplastic filament that is usually 1.5–3 mm in diameter. The filament is heated up to the temperatures at which the used polymer experiences technological softening; then, the surfacing head moves along a given trajectory and the softened polymer filament is extruded through the nozzle and applied onto a special platform and, then, layer by layer to the previous layers according to a preliminary prepared 3D-CAD model. As a result, a three-dimensional product is formed.

The main technological parameters of the FDM method are as follows:

- Extruder temperature, which is set depending on the softening or melting temperature of the polymer used;
- Layer thickness, which is associated with the diameter of the used polymer filament;
- The speed of the extruder nozzle motion;
- Infill rate, which can be less than 100% in cases where it is needed to obtain a porous structure;
- Printing strategy;
- Method of attaching the first layer to the build platform;
- Build platform temperature.

**Figure 10:**
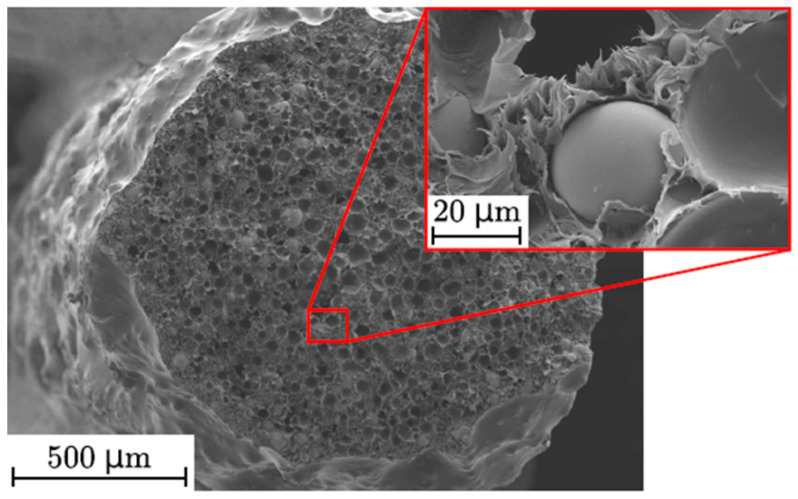
SEM image of a filament with spherical NdFeB particles in a polyamide (PA11) matrix [41].

By using the FDM method, it is possible to fabricate products with a high degree of detail with a spatial resolution of up to 500 µm and with a fairly low roughness Ra = 12.4–23.7 µm [68]. This method is quite well studied with respect to polymers, but the addition of a magnetic filler to the polymer matrix introduces quite a lot of nuances into the printing process. For FDM printing of magnetic materials, a pre-prepared composite filament is usually used (Figure 10). To prepare it, single or twin screw extruders are used [71,72]. The production of composite filaments can be carried out by any other manufacturer [41,58] or directly in the FDM printer with the connection of such an extruder to it [48,71,73]. To obtain a uniform distribution of the filler in the filament, various techniques are used. For example, in [71], cooling of the manufactured primary composite filament in liquid nitrogen is used to effectively grind it into a powder and to use this composite powder to manufacture the finished composite filament. 

There are some technological features of fabricating magnetic polymer matrix composites. To make the composite a magnetic one, a metal or ceramic powder is usually used as a magnetic filler. Such a filler has a hardness and an elastic modulus that significantly exceed those of polymers, which leads *to a notable reduction in the composite viscosity* that can turn into printing difficulties. In particular, filament breakage, as well as nozzle clogging, may occur during the printing process. The most commonly used thermoplastics in FDM printing are polylactides (PLA), acrylonitrile butadiene styrene (ABS), polycarbonate (PC), and polyethylene terephthalate (PET) [68,74]; however, to facilitate the production of composite materials, more specific types of polymers are also being developed, such as those with higher flow and better shaping [74]. Such properties, for example, are possessed by various polyamides (PA), also known as nylons and polyoxymethylene (POM) [68]. Other polymer matrices such as polyether ether ketone (PEEK) may also be used [75]. To further increase the fluidity of the composite and to ensure a uniform distribution of particles in the matrix, a high molecular weight dispersing agent was also used in [68]. It was added in an amount of 3% based on the weight of the filler powder. To improve the fluidity of the powder and to reduce friction between particles, a small amount of fumed silica was added as a lubricant. 

Various approaches can be used to reduce viscosity. In addition to adding special additives, it is possible to slightly increase the nozzle temperature for greater softening of the polymer component [76]; however, in this case, it is necessary to choose a temperature that will not lead to a degradation of the polymer. At the same time, *when a composite filament is heated, degradation of the polymer matrix can be observed at lower temperatures than in the case of the same polymer filament without a filler*. It can be explained by the rather high thermal conductivity of the filler particles compared with that of the polymer, and the polymer is heated more intensively in this case [75]. It should be taken into account when choosing the nozzle temperature if it is rather close to the polymer degradation temperature.

The main characteristics of the powder particles that affect the viscosity are their shape, size, and volume or mass content. The shape and size of the powder particles usually depend on the method of their preparation. Here, several main groups of powders that are used in FDM technology can be distinguished. They are spherical powders with a particle size of about 30–50 μm, obtained mainly by gas atomization [30,60,61,68]; powders with irregular, flaked, or fragmented shaped particles with the longest dimension from several microns to several millimeters obtained by mechanical grinding in ball mills [77] or by melt spinning [78,79]; and micron and nanosized particles of various shapes obtained by chemical methods [76]. *The spherical shape and fine size usually promote decreased viscosity.* For injection molding (IM), which is a conventional technology for manufacturing permanent polymer-bonded magnets, the most optimal filler is spherical particles with a size of about 45 μm [41,80].

With an increase in the content of filler particles in the polymer matrix, its viscosity increases significantly. For example, in [72], with an increase in the content of a steel powder in the ABS matrix from 10 vol.% to 40 vol.%, the viscosity increased from 1.22 kPa·s to 4.02 kPa·s. As a rule, the volume content of the filler is from 45% to 65% [41]. In mass terms, the proportion of particles can reach 80 wt.%. *However, each polymer matrix has its own critical value of the filler content, above which the high brittleness of the composite filament takes place* [68]. For example, in [30], polyethylene (PE) only partially covered the filler particles when the filler content was of 86.5 wt.% and more. With a large number of filler particles, the total bond between the particle surface and the matrix degenerates, which also leads to a deterioration in mechanical strength. In general, the adhesion between the metal particles of the filler and the polymer matrix is very low [73], which can also be observed in photographs of the cross section of the filament, for example, as in Figure 10. Some particles are located separately in the viscous pits of the polymer. If the content of filler particles is too high, the amount of polymer binder is insufficient. It is caused by the existence of a maximum level of powder material compactability, which is always lower than that of a bulk counterpart. In addition to very low adhesion, it also leads to an increased porosity compared with composites with a lower filler content [73]. The authors of [73] also explained this porosity using the fact that *there is a faster cooling of the deposited filament due to the presence of a metal powder with good thermal conductivity*. Porosity leads not only to the deterioration of mechanical properties but also *to internal oxidation of the magnetic powder* when air or moisture penetrates into the pores, which is especially critical for magnetic materials containing iron and having low corrosion resistance, which will eventually lead to degradation of the magnetic properties. *Due to the low adhesion to the polymer, some metal powder particles come out of the filament and are present directly on the surface of the deposited layer, inhibiting good polymer binding (adhesion between layers).* In some cases, the particles themselves act as structural defects where destruction occurs.

In [79], in order to improve the adhesion between the polymer matrix and the metal filler, 1 wt.% of titanium triisostearoylisopropoxide (TTC) was added to the NdFeB-PA12 composite precursor as a binding agent [81], 0.2 wt.% of zinc stearate was also added as an external lubricant, and 0.5 wt.% stearic acid was added as an internal lubricant; these substances reduce friction between particles and protect the nozzle from wear [82].

The presence of a filler can affect the glass transition temperature of the filament, which leads to an influence on the technological softening of the polymer. In the presence of a filler, the glass transition temperature slightly increases compared with the same polymer filament without a filler; the shift is up to 25 °C [75].

*The low content of the filler in the composite can also negatively affect the quality of the product.* Thus, in [30], the PE matrix began to agglomerate at a filler content of less than 63.1 wt.%, which results in an uneven distribution of the filler.

**Figure 11:**
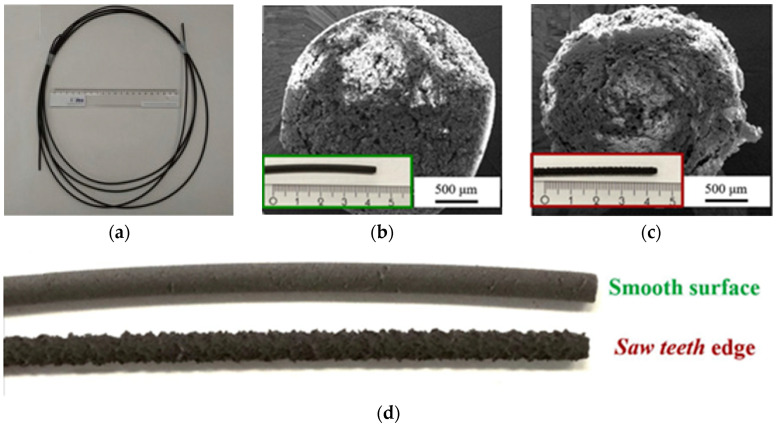
(**a**) Extruded MnAlC (80 wt.%)—ABS filament (a 20 cm ruler is included for scale comparison); (**b**) SEM images of the circular cross section of MnAlC—ABS filaments with different-weight fine-to-coarse particle ratios (FP/CP) 25/75 and (**c**) 50/50. Insets show images taken of the corresponding filaments for comparison of the filament surface; (**d**) closer view of the filaments in (**b**,**c**) for detailed comparison between the smooth surface obtained with the optimal FP/CP ratio and the irregular surface obtained for a non-optimal FP/CP ratio [83].

The introduction of *a powder with several modes in the particle size distribution* into a composite is considered *an effective way to reduce the viscosity and to increase the possibility of introducing more filler into the composite.* Thus, in [83], fine and coarse powders with average particle sizes of 16 and 30 μm, respectively, were introduced in various ratios. A significantly improved quality of the composite filament surface was observed when introducing a higher content of fine particles at the same total filler content (Figure 11), which indicates the potential of introducing more filler with the approach of using bimodal powders. In [79], it was also reported that the use of spherical particles provides a smoother filament surface than in the case of irregularly shaped particles with the same content.

**Figure 12:**
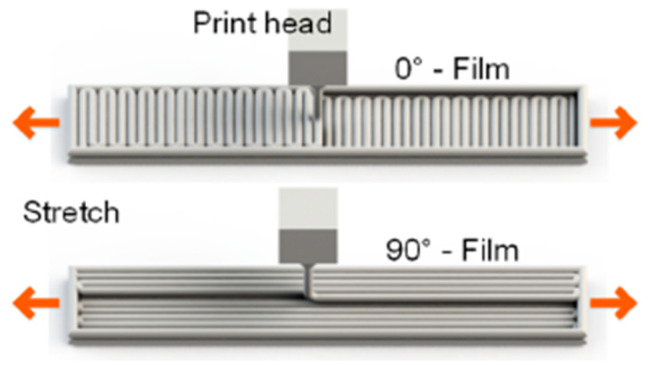
Schematic representation of the printing strategy [71].

The FDM printing strategy mainly influences the mechanical characteristics of the manufactured product. For example, in [84], it was shown that, under standard tensile tests, specimens with the filament laid along the tensile direction always had better mechanical properties, including a higher modulus of elasticity, than specimens with the filament directed perpendicular to the tensile direction (Figure 12). The authors of [84] explain this fact using the higher adhesion between the filament sections in the first case. In the second case, the total contact area between the filament sections is larger, which results in a larger number of pores as they are formed between the filament sections. An increase in porosity always leads to a general decrease in mechanical properties. However, an increase in the number of layers promotes some increase in the elastic modulus, which can be explained by partial filling of voids by melting of the previous layer when applying the next one. 

When applying FDM-type technologies, a parameter such as platform heating can be effectively used. On one hand, *it allows for the polymer matrix to dry by evaporating the moisture contained within and to achieve faster curing*, and one the other hand, it helps *to reduce the temperature gradient*. When depositing filaments onto a cold build platform, the first layers are cooled much faster than those farther from the platform. It is important for amorphous-crystalline polymers because of their different degrees of crystallinity. For layers near the platform, it is less than that for distant layers with a lower cooling rate. It leads to the formation of an inhomogeneous structure, which gives anisotropy of mechanical properties and their general deterioration. The most used platform temperatures usually lie within the range from 45 °C [85] to 110 °C [72] depending on the type of used polymer matrix.

**Figure 13:**
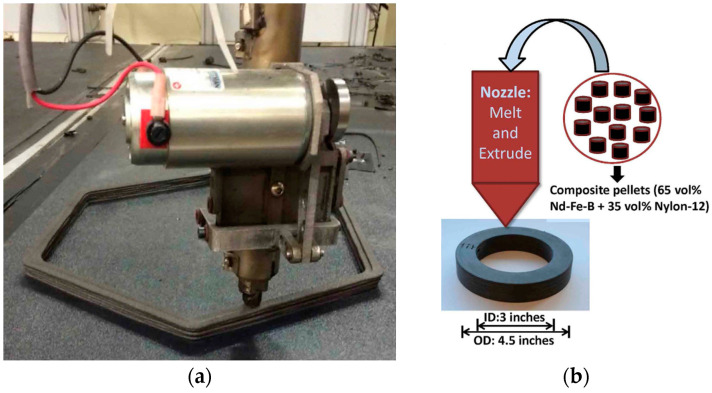
(**a**) Image of the nozzle depositing layers of magnetic materials on the print bed for BAAM technology; (**b**) schematic of the melt and extrude process, in which right underneath the nozzle is a printed magnet in a hollow cylinder shape with an OD × ID of ~4.5 inch × 3 inch [86].

One of the varieties of FDM technology is Big Area Additive Manufacturing (BAAM) [86] (Figure 13), *which makes it possible to overcome the disadvantage of typical FDM technology, which is the limited size of manufactured parts*. The BAAM process does not require pre-fabrication of the filament. The starting material for BAAM technology is composite granules. They are produced by high-energy ball milling a mixture of feedstock polymer pellets and a filler powder, followed by extrusion.

Similarly to FDM, one of the disadvantages of BAAM is *the presence of up to 20% porosity in printed products* due to the rather rapid cooling of the polymer with insufficiently tight fit of the deposited filament to the previous layer [86]. However, *tailoring the used technological parameters and materials can lead to good prospects* for using this method for manufacturing complex functional products of different shapes and sizes.

### 3.1. Magnetic Materials for ATs

Magnetic materials are usually divided into two technical groups: hard magnetic and soft magnetic materials. This division depends on the values of coercivity H_c_. Hard magnetic materials should have H_c_ > 1 kA/m (12.6 Oe), and soft magnetic materials should have H_c_ < 1 kA/m (12.6 Oe) [39]. However, this value is rather conditional, and it may slightly vary between different papers. The principle of this division is generally based on the application fields of the magnetic materials.

Hard magnetic materials are usually used for manufacturing permanent magnets of various configurations for sensors, particle accelerators, parts of electric motors, electric drives, etc. They should have not only high H_c_ but also high remanent magnetization Br and high maximum energy product (BH)_max_, providing high magnetic energy of the magnet, which can be converted into mechanical performance.

**Figure 14:**
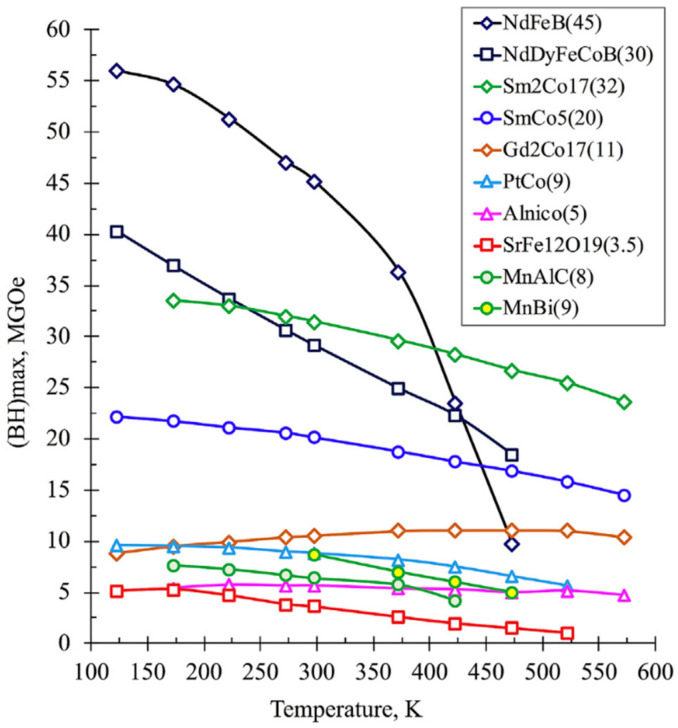
Temperature dependence of (BH)max for most commercial permanent magnets. The value in parentheses in (BH)max at 298 K [87].

NdFeB-based magnets currently have the highest energy product values of any known hard magnetic material and are widely used (Figure 14). Due to this fact, they are still among the most studied hard magnetic materials, including additively manufactured ones. Despite very high magnetic properties, NdFeB-based magnets also have a number of drawbacks. They have not very good mechanical properties, a tendency to oxidize, and low operating temperatures. For this reason, other compositions are being developed in order to increase chemical and temperature stability. Figure 14 shows the temperature dependences of the energy product of various materials according to [87]. From this figure, it can be seen that, at temperatures above 170 °C, it is more reasonable to use Sm-Co-based magnets, although such magnets also have not very good mechanical properties with a rather high brittleness. For this purpose, the well-known Alnico magnets, as well as exotic compositions such as Gd-Co, Pt-Co, Mn-Al, etc., can also be utilized [87].

Hard magnetic ferrites show the lowest magnetic properties among various hard magnetic materials; however, polymer-bonded ferrite magnets are ideal for applications where an inexpensive magnet combined with a complex shape of high precision is required [88]. In [88], the use of both materials, *SrFe_12_O_19_ ferrite and NdFeB alloy*, was proposed to obtain magnets with *a good combination of properties and cost,* since rare earth magnets are among the most expensive ones.

New compositions are also being actively developed and studied. *In particular, a rather crucial task is the development of modern permanent magnets that do not contain rare earth elements* [87,89,90,91]. In [30,83], the preparation of magnets based on MnAlC ((Mn_57_Al_43_)_100_C_1.19_) alloy with PE [30] or ABS binder [83] was studied. The coercivity of such magnets remains almost constant after fabrication processes with a value of about 1.53 kOe for both the composite and the filament at a filler content of 72 wt.%.

*Cerium-based magnets* have attracted great attention both in academic and industrial fields due to their *good combination of a low cost and the possibility to obtain phases with excellent magnetic characteristics* [92]. Cerium-based sintered magnets have widely adjustable magnetic properties with varying Ce content. The intrinsic coercivity H_ci_ of such magnets is from 80 to 2400 kA/m, and (BH)_max_ is from 230 to 400 kJ/m^3^, so they can meet many different requirements and can be used in various fields, from the packaging market to motor drive production, etc. Ce-containing magnets doped with Co show excellent thermal stability, which is promising for their use in electric or hybrid car engines in the near future. 

Soft magnetic materials are mainly used for the manufacture of transformer cores of various configurations, magnetic conductors, antennas of microwave elements, magnetic and electromagnetic shielding, and so on [39,67]. Soft magnetic materials should have low H_c_, which ensures minimal hysteresis losses, since with an increase in the operating frequency, the total losses become very significant even at a small increase in the coercivity [39]. When remagnetizing with a sufficiently high frequency, eddy current losses become very noticeable. It results in a necessity to use magnetic materials with high electrical resistance for high-frequency applications. This is often achieved by introducing an insulating material to the magnetic material using various methods. For example, stacked cores of transformers are made from thin plates of electrical steel with interlayers of insulating material. Metal–polymer composites made with magnetically soft particles distributed in the polymer matrix can also be used. However, for low-frequency applications, it is recommended to use materials with low electrical resistance to reduce heat losses. Soft magnetic materials should also have low B_r_ and generally low remagnetization losses, as well as a number of additional characteristics such as high values of initial and maximum permeability at low and high frequencies, and a high level of saturation magnetization B_s_ is preferable. 

Coercivity is a property that is extremely sensitive to the structural state of a material. One of the main characteristics of a polycrystalline material structure is its grain size. Herzer [93] showed an inversely proportional dependence of the coercivity on the grain size down to grain sizes of 100 nm. The dependence is generally linear. It can be explained by an increase in the number of grain boundaries that pines the motion of domain walls during magnetization. When the grain size decreases to smaller than 100 nm, the coercivity begins to decrease sharply, which is associated with the start of dominant exchange processes and a change in the mechanisms of macromagnetic properties formation. *The size of the individual particles of a magnetic filler affects the coercivity in the same way as the grain size in a bulk polycrystalline material*. It is worth noting that micron-sized particles can also possess an internal structure with nano-sized structural elements, and in this case, the coercivity mainly depends on the size of the nano-sized structural element. Such powders can be considered nanostructured ones, and they are actively studied as promising magnetic materials but will not be described in this review.

### 3.2. Fabrication of Hard Magnetic Materials by FDM

As mentioned above, magnets based on NdFeB alloy are among the most studied in the field of their production by AT methods. When using polymer technologies for the manufacture of magnetic materials, an important factor that influences the magnetic characteristics is *the volume fraction f of the filler powder in the polymer matrix*. First of all, it affects *B_r_*. The more magnetic filler is contained in the polymer matrix, the higher the remanent magnetization is, since a pure polymer is a non-magnetic phase. Moreover, the dependence of B_r_ on the filler content is almost *linear*.

Coercivity does not usually depend so dramatically on the volume content of the filler; *it is a function of the structural state of the filler itself*. The heating used in the *FDM* method is relatively insignificant, and the effect of temperature is rather short, so *the structural state of the magnetic fillers in this technology does not noticeably change*. As a result, the composite has the same internal coercivity as the feedstock powder. At constant H_c_ and varying B_r_, the energy product (BH)_max_ also changes, which, is a function of the volume fraction of the filler as well. Moreover, it is proportional to *f*^2^ [40]. There are also papers reporting that *H_c_* can significantly depend on *f* as well as B_r_ does [75].

Since the polymer, in particular, in FDM filaments, rarely contain more than *60 vol.% of magnetic fillers* [41], the remanent magnetization is approximately half of the remanent magnetization of a sintered magnet of the same composition without a polymer binder. At the same time, it can be noted that the properties of magnets fabricated by polymeric ATs and those of polymer-bonded magnets fabricated by conventional IM are *comparable*, and in some cases, the former may even be *slightly higher*. Using the NdFeB alloy as an example, Table 1 compares the magnetic properties of the magnets fabricated by different technologies. Along with this, the use of polymeric ATs opens up more opportunities for fabricating various complex structures *without the need for new tooling*. 

In addition to NdFeB magnets, ATs are also being explored for manufacturing magnets with other compositions or even *mixtures of several known compositions*. In [102], composite pellets were used. They contained 65 vol.% of anisotropic powders Nd-Fe-B (Nd_2_Fe_14_B) + Sm-Fe-N (Sm_2_Fe_17_N_3_) and 35 vol.% of Nylon 12. These pellets are usually used for conventional IM of anisotropic magnets. The pellets were used as the feedstock material for 3D printing using the BAAM method. In the as-printed state, the samples had the following properties: B_r_ = 370 mT and H_c_ = 10.7 kOe, the same as the original composite pellets. In general, the properties changed with respect to the properties of the magnetic powder according to the same laws as described above.

The authors of [103] proposed combined structures based on hard magnetic (BaFe_12_O_19_) and soft magnetic (NiFe_2_O_4_) ferrites, fabricated by extrusion-type 3D printing implemented on the basis of an FDM printer. In this work, inexpensive oxide powders (NiO/Fe_2_O_3_ and BaCO_3_/Fe_2_O_3_) were used as precursors. They were first used for paste preparation by mixing them with a water-based binder and a plasticizer. The binder was polyvinyl alcohol (PVA), and the plastisizer was polyethylene glycol (PEG-400). The samples printed from this paste were subjected to debinding and sintering at temperatures of 1000–1400 °C. At these temperatures, high-temperature synthesis of the ferrites of the required compositions occurred. With an increase in the sintering temperature, the saturation magnetization of ferrites increased. Thus, for NiFe_2_O_4_ ferrite, it increased from 38.32 emu/g after sintering at 1000 °C to 48.39 emu/g after sintering at 1350 °C. The authors of [103] attribute this to a significant grain growth, as well as to a decrease in the defectiveness of the structure during a long holding time, about 60 h, at high temperatures. However, the highest value of saturation magnetization was lower than that of bulk samples of this compound, for which it was 56 emu/g. The highest saturation magnetization for ferrite BaFe_12_O_19_ was 64.94 emu/g, which practically reached the value for the bulk ferrite, which was 67.7 emu/g.

### 3.3. Fabrication of Soft Magnetic Materials by FDM

Soft magnetic materials, in contrast to hard magnetic materials, should have a minimum coercivity, minimum hysteresis losses, high saturation magnetization, and maximum values of permeability. At the moment, the most widely used soft magnetic materials are pure iron [39,104], electrical steels based on an Fe-Si system, permalloys [8], soft magnetic ferrites such as Fe_2_O_3_, Fe_3_O_4_ [66,105,106], NiZn [101], and amorphous and nanocrystalline alloys based on cobalt and iron [8,33,107,108,109,110].

The use of polymeric ATs allows for fabricating a product of almost any shape from magnetically soft materials *with an already existing insulating material in one operation*. It provides a great advantage to AT in the field of manufacturing of high-frequency electromagnetic devices, in particular, various cores for transformers. Similarly to hard magnetic materials fabricated by polymeric ATs methods, the main factors that influence the magnetic properties are *the shape and size of the soft magnetic particles, and their content in the polymer matrix*. For example, *larger particles can lead to an increase in magnetic properties due to less insulating material covering the particles* [111]. As for B_s_, it *linearly* depends on the filler content in the matrix [71,84].

**Figure 15:**
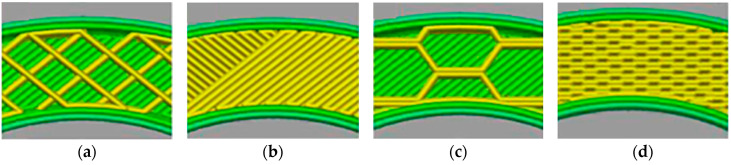
Horizontal cross sections of transformer cores modeled on Simplify3D with different fill patterns and percent fill: (**a**) rectangular fill pattern with 20% fill; (**b**) rectangular fill pattern with 100% fill; (**c**) honeycomb fill pattern with 20% fill; (**d**) honeycomb fill pattern with 100% fill [67].

In [67], transformer cores were fabricated using the FDM method from a composite filament containing 40 wt.% of irregularly shaped Fe particles in a PLA matrix. Such a filament showed the saturation magnetization of 69.69 emu/g and the coercivity of less than 10 Oe, which generally meets the criteria for soft magnetic materials. In this work, the influence of infill rate and the strategy of printing on the magnetic characteristics was also studied. The internal structure was printed in the form of rectangles (Figure 15a,b) or honeycombs (Figure 15c,d). As one would expect, the lower the infill rate is, the lower the magnetization and permeability are, and a higher field is required to magnetize such a core. In [67], the printing strategy did not affect the shape of the magnetic hysteresis loop.

In [85], the FDM preparation of a soft magnetic composite based on a PLA matrix with 35 wt.% of iron particles was studied. Ethylene vinyl acetate (EVA) was used *to improve adhesion to the substrate*.

In [72], the FDM preparation of a magnetic composite based on 40 vol.% of an Osprey 17-4PH stainless steel filler in an ABS matrix was studied. The resulting samples had the following properties: B_s_ = 403 mT, B_r_ = 15.6 mT, H_c_ = 6.51 kA/m. In this case, the initially soft magnetic material in the form of a composite behaved like a *hard magnetic material*, which makes it promising as an application for the manufacture of *relatively inexpensive sensors and actuators*.

### 3.4. Magnetic-Field-Assisted ATs

The magnetic properties of a material working in a structure are formed as a result of a certain pattern of magnetization distribution which is formed under the influence of many factors, such as atomic structure, the presence of crystallographic anisotropy, external and internal mechanical stresses, and the shape of the structure. In magnetic powders of a given chemical composition that are used for structure fabrication, the main factors are crystallographic anisotropy and shape anisotropy. *Crystallographic anisotropy* is a characteristic of *all crystalline* magnetic particles, since in such particles, there are crystallographic easy magnetization axes. In an external magnetic field, the particles tend to align with the magnetic field. *Shape anisotropy* is inherent in *irregularly shaped* magnetic particles, in which there is the longest dimension, since it is always more profitable for such particles to be magnetized exactly along this direction. In this case, the longest dimension of the particles tends to align with the magnetic field.

Theoretical and practical aspects show that in order to *improve* the magnetic characteristics of magnetic materials, it is necessary to create the *maximum magnetic anisotropy* in a structure [39,76,112]. According to the Stoner—Wohlfarth model, B_r_ and H_c_ decrease by a factor of 2 in the case of randomly oriented single-domain particles compared to completely aligned single-domain particles [113]. To obtain magnetic anisotropy, an *external magnetic field is often applied during the manufacture of magnetic materials or their post-processing* to align the magnetic particles with the desired direction in the printed product. This approach is applicable to both hard and soft magnetic materials with a difference in the required direction of the particles’ orientation. Thus, for soft magnetic materials, the optimal state is when the particles in the product are aligned with the directions of easy magnetization along the external magnetic field. In contrast, for the hard magnetic materials, it is more advantageous when the particles are aligned with the directions of hard magnetization along the external magnetic field.

In [76], an anisotropic magnetic structure was formed in composites based on the plate-like particles of strontium hexaferrite and spherical particles of Sm_2_Fe_17_N_3_ in a PA6 or PA12 matrix under a magnetic field action. As a result, the remanent magnetization in two directions of the printed magnets based on strontium hexaferrite was 222 mT and 201 mT and the coercivity was 207 kA/m and 162 kA/m. As for magnets based on Sm_2_Fe_17_N_3_, these values were 389 mT and 308 mT, and 899 kA/m and 565 kA/m, respectively, which indicates the presence of anisotropy in the resulting magnets. With regard to the magnetic particles of strontium hexaferrite, they have shape magnetic anisotropy. Thus, in the plate-like particles of strontium hexaferrite, the axis of easy magnetization is oriented perpendicular to the longest side of the plate. Spherical Sm_2_Fe_17_N_3_ particles do not have shape magnetic anisotropy, but they are oriented in a magnetic field in accordance with their crystallographic easy magnetization axes.

The ATs can also be upgraded *using an external magnetic field source integrated into a 3D printer*. An electromagnet in the form of a solenoid connected to an external current source can serve as a field source. In this case, the intensity of the external magnetic field is controlled by changing the current in the coil. In general, the magnetic field strength depends not only on the electric current value but also on the size and configuration of the coil. This means that it is theoretically possible to create an electromagnet that provides any desirable magnetic field strength. However, when applying a *too-strong magnetic field*, the product under its effect can be *deformed* in the process of printing. The critical value of the external magnetic field strength is determined primarily by the magnetic characteristics of the *filler particles*, in particular, by their *remanent magnetization*. The *viscosity* of the polymer matrix also contributes to its level [76].

**Figure 16:**
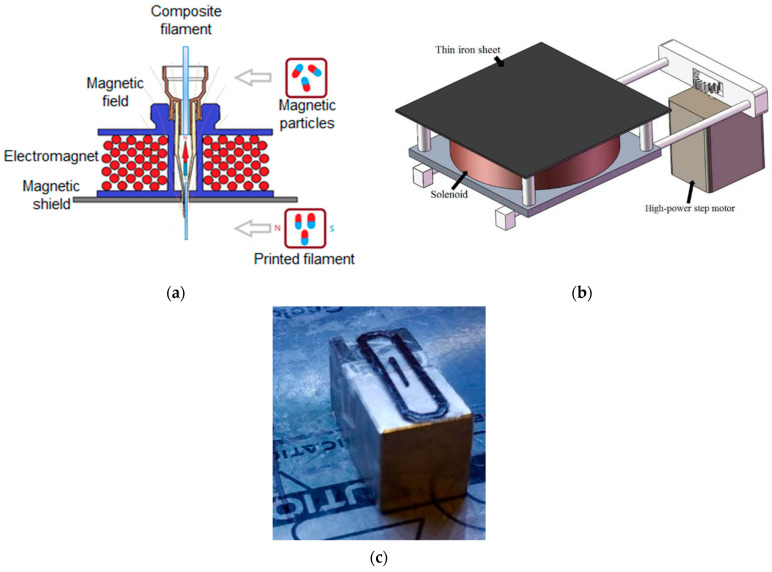
(**a**) The ferromagnetic particles embedded in the composite filament are reoriented by the applied magnetic field generated by an electromagnet placed around the dispensing nozzle adopted from [114]; (**b**) by using a special building platform with an electromagnet placed under it [95]; (**c**) or by using a permanent magnet [85].

This coil can be placed into the printer in such a way that the nozzle is located inside it along the central axis (Figure 16a) [114]. In the nozzle zone, the heated polymer is in a low-viscosity state, and it is easier for the magnetic particles of the filler to align with the direction of the external magnetic field. In this case, the lines of the magnetic field pass along the filament and the magnetic particles are aligned along the filament as well.

In [95], a special magnetic platform was developed (Figure 16b). An electromagnet solenoid was placed under a thin iron sheet on which the product was built. A powerful stepper motor was also attached to the platform to ensure printing stability and dimensional accuracy. The induction of the magnetic field was set to values of 0.3 T, and under the influence of this magnetic field, NdFeB-based magnets with a polycaprolactone (PCL) polymer matrix were printed.

An external magnetic field can be provided not only by *electromagnets* but also by *permanent magnets*. For this purpose, NdFeB magnets in the form of cubes or bars are usually used [76,85] (Figure 16c). In this case, the strength of the external magnetic field can be controlled by changing the distance between the magnet and the applied track [70]. It is possible to change not only the strength of the magnetic field but also its direction by changing the position of the magnets in space.

In [85], magnetically assisted FDM printing of a soft magnetic composite based on PLA with iron particles led to the anisotropy of the magnetic susceptibility along the easy and hard magnetization axes χ_easy_/χ_hard_ = 2.7. This rather high value allows magnetic-field assisted FDM technology to be used for printing *efficient transformer* cores with good coupling between the primary and secondary coils, as well as to develop a new approach to manufacturing various *sensors and actuators with competitive characteristics*.

In addition to applying an external magnetic field directly during the printing process, some papers indicate that magnetic post-processing can be carried out as well. In [102], magnet samples based on a Nd-Fe-B + Sm-Fe-N mixture were subjected to an external magnetic field with a strength of up to 4000 kA/m after printing. Since the viscosity of a cooled thermoplastic is rather high after printing, it makes it difficult to orient particles even in a strong magnetic field. To overcome this issue, the authors of [102] suggested *heating* the printed product above the glass transition temperature of the matrix to soften it. It is important not to overheat it up to thermal decomposition temperatures. In this paper, even in a field of 400 kA/m, the remanent magnetization significantly increased with an insignificant decrease in the coercivity. For example, after treatment in the field of 1600 kA/m, the coercivity was 812 kA/m and the remanent magnetization was 730 mT, while the maximum energy product was 89.9 kJ/m^3^ (11.3 MGOe); a further increase in the magnetic field strength did not practically lead to any change in these properties. However, the application of such strong fields in the printing process can be very difficult. The authors of [102] proposed an approach to use *pulsed* fields in the printing process, as well as to use fields with a lower strength of 800–1200 kA/m, which may be sufficient in some cases. 

## 4. Direct Ink Writing

**Figure 17:**
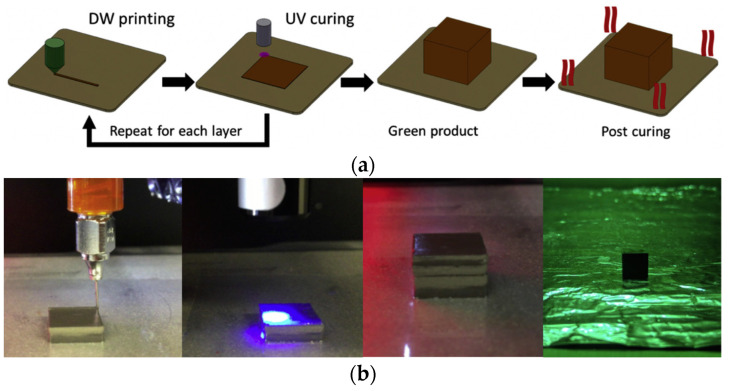
(**a**) Scheme of the UV-assisted direct writing (UADW) process; (**b**) actual images of the process for printing a cube-shaped magnet [78].

The DIW technology principle is similar to the FDM technology. A soft polymer or composite is applied through a nozzle to the build platform and then layer by layer to the previous layers according to the 3D-CAD model and the specified scanning strategy. However, in the case of DIW, *low-viscosity* polymers are used *without filament pre-fabrication*. *Due to the low viscosity, surface tension can promote better bonding of the layers and minimize or eliminate pores in the product and to achieve good product detail.* In addition, the low viscosity of the used polymers makes it possible to introduce up to *98 wt.%* of the filler [115]. At the same time, the low viscosity results in the fact that the printed products do not keep their shape even when a filler is introduced [115], which is why additional energy sources are used for their curing. They are often special UV lamps with a small focal spot. Figure 17 shows the scheme of DIW method given in [78].

In [78], the first layer was deposited onto a transparent polyimide substrate; then, this layer was treated with a UV source, which moved along the same trajectory as the print head. To ensure a narrow focal spot, the source must be located at a certain distance from the layer being cured. UV curing of the layer occurred before applying the successive layer. After printing, the product was turned over with the substrate and additionally exposed to UV radiation through the polyimide substrate. The sample separated from the substrate was subjected to additional UV treatment using high-intensity radiation with different wavelengths. It was followed by drying at temperatures of 60–80 °C for its complete curing.

In DIW technology, various photocurable resins, such as oligomers and monomers of methacrylate [78], are often used. In the initial state, they have a very low viscosity. Similarly to FDM technology, this low viscosity significantly increases when introducing a powder filler into the polymer matrix. It can also lead to difficulties or even impossibility of printing due to the mixture getting stuck in the nozzle. As a rule, the nozzle size should be at least *ten times* bigger that the characteristic particle size [116] in order to avoid clogging and hindering of the printing process.

If there is significant shape anisotropy of the particles used, their *orientation* affects the total viscosity of the mixture [117]. Theoretically, particles with a large aspect ratio will gradually align along the shear plane as the shear rate increases, resulting in reduced viscosity and shear thinning [78,118]. The viscosity of suspensions with irregularly shaped particles is usually higher than that of suspensions with spherical particles [97]. This can be explained by the increased interactions between the particles due to their irregular shape, since they are randomly distributed in the suspension and form a large number of contacts with other particles during shear flow [78].

There are several models for estimating viscosity, for example,

- Model proposed by Mooney [119]

(9) $$\eta_{r}=exp(\frac{kf}{1-\frac{f}{f_{m}}}),$$

where *η_r_* is the relative viscosity, *k* is a constant depending on the particle form factor, and *f_m_* is the maximum volume fraction of particles in the suspension. For ideally spherical particles, it is assumed that *k* = 2.5; for irregularly shaped particles, this constant becomes empirical and has a value greater than 2.5, up to 15. The value significantly depends on the particle form factor and the surface roughness, and it is determined experimentally [78]:

- Model proposed by Krieger and Dougherty [120]

(10) $$\eta_{r}={\left(1-\frac{f}{f_{m}}\right)}^{-kf_{m}}$$

Calculations according to these models, when using monodisperse spherical particles with a unimodal distribution, show that the maximum volume fraction of particles should not exceed 0.69. It is generally similar to the maximum allowable content of filler particles in FDM filaments. To increase the volume fraction of introduced filler particles at acceptable viscosity values, in [121,122], the introduction of a powder with a bimodal size distribution was proposed. At a certain size ratio of smaller particles to larger ones, the former will fill the gaps between the large particles, thereby maximizing the total volume fraction of the filler.

In [78], by using irregularly shaped particles, it was shown that there is a *nonlinear* dependence of the viscosity on the parameter ζ = f_1_/f, where f_1_ is the volume fraction of the coarse fraction and f is the entire volume fraction of the powder. There is a value of parameter ζ, of about 0.5–0.7, at which the lowest viscosity is observed. The dependence of viscosity on the ratio of the larger dimension of the particle to the smaller one is also nonlinear, and there are optimal values of such a form factor. For example, in [78], the optimal value of the form factor was 16. However, despite the fact that in [78], *the bimodal size distribution made it possible to increase the maximum volume fraction of the powder*, nevertheless, *this led to a distortion in the shape of the printed product and its rougher surface*. The use of coarser powders requires the use of larger diameters of nozzles, which in turn results in a thicker layer and a rougher product surface. 

In [123], the preparation of a composite based on anisotropic iron particles in a photoresist based on epoxy resin was studied. In DIW technology, as well as in FDM technology, nozzle size is important in ensuring high-quality printing, which should also be related to the maximum particle size used. Another parameter that affects print quality is print speed. The higher it is, the thinner the resulting tracks and the higher the degree of detail are. However, if the print speed is too high, the track may be destroyed directly during the printing process. *Print speed should be related to the viscosity of the used ink,* which increases with an increase in filler content. *At the same time, an increase in viscosity leads to a certain increase in the degree of detail, since if the viscosity is too low, the track begins to blur, and the product loses its accurate shape*. In this method, there is also an optimal filler content of about 40 vol.%, which allows for obtaining a sufficiently high quality printed product and not damaging the equipment as too much filler content leads to a very high viscosity of the composite and clogging of the nozzle. To reduce the pressure of the composite ink on the nozzle and to reduce the friction between it and the particles, in [123], 1% acetone was added to the ink in order to thin it. In addition, before printing, the nozzle tip was dipped in oleic acid, which acted as a lubricant.

In [77], the preparation of a composite based on Fe_16_N_2_ flake powder in a photopolymer matrix was studied. Fe_16_N_2_ is a compound with a giant saturation magnetization that is close to or exceeding that of rare earth permanent magnets. The abundance of its elements and the low cost of synthesizing this compound made it very attractive in terms of the replacement of permanent magnets based on rare earth elements. The synthesis of the compound can be carried out by nitriding pure iron particles in an ammonia atmosphere. In [77], when printing using the DIW method, an external magnetic field of 0.7 T was applied. As a result, the composite had the following properties: H_c_ = 350 Oe, M_s_ = 106 emu/g with a filler content of 50 wt.%.

In [124], an experimental setup was developed to study the orientation of irregularly shaped hard magnetic particles of strontium ferrite in a photo-sensitive polymer matrix using an external magnetic field generated by a pair of permanent magnets. As a result of the interaction between magnetic particles, chain microstructures were formed. A significant increase in the number of oriented structures with decreasing distance between permanent magnets was demonstrated, and it was possible to increase the initial magnetization of the particles. The interaction between particles and the formation of *chain microstructures* during the initial stage of magnetization were significant and provided rotation of the resulting chains even at low magnetic flux densities.

In [115], the preparation of a composite based on NiZn ferrite was studied. The UV-curable matrix consisted of pentaerythritol tetraacrylate (PETA), which is a UV-sensitive monomer, and a photoinitiator. Polyethylene glycol (PEG) was also used as a diluent to increase the flowability of the paste. Since the ferrite powder was quite fine, with an average particle size of 1.1 μm, in this work, it was possible to achieve a content in the composite up to *96 wt.%*. At the same time, it was noted that, with an increase in the filler content, the effect of UV treatment *decreased* and the printed products were cured poorly. When introducing more than 96 wt.% of the filler, there was *practically no curing*. After printing, the samples were also subjected to debinding and sintering to improve the mechanical properties. As a result, typical models of toroidal and E-shaped transformer cores were fabricated.

Since ferrite cores are often used in microwave engineering, the authors of [115] studied the frequency magnetic characteristics of the printed samples. After sintering, the real part of permeability was 90 in the frequency range from 0.1 to 15 MHz. and the peak frequency was 37 MHz. It even *exceeded* the values for a commercial counterpart [115]. The permeability was similarly to magnetization directly proportional to the filler content in the matrix.

One of the varieties of DIW technology is *inkjet printing (IP)*. The difference is in using polymer-based pastes with even *lower* viscosities for IP than for DIW. In IP technology, in addition to the mode of continuous deposition of an ink, the mode of dropwise deposition is implemented. Here, there is also the possibility of *multimaterial* printing using several feeders in inkjet printers [70]. *Due to an even higher degree of detail, as well as due to the low productivity of this method, it can be utilized for the deposition of functional coatings and for the manufacture of miniature products from magnetic materials.*

In [8,125], the possibility of using the IP method for manufacturing toroid cores based on pastes from a trimethylol propane triacrylate (TMPTA) monomer solution and a mixture of Permalloy and Metglas 2705M powders was shown. The possibility of fabricating cores based on NiZn [125] and NiCuZn ferrites in matrices based on an aqueous solution of methylacrylamide and N,N′-methylenebisacrylamide [125], and pentaerythritol tetraacrylate with phenylbis(2, 4, 6-trimethylbenzoyl)phosphine oxide as a photoinitiator was also shown [8]. Ferrite-based cores after printing were subjected to debinding and sintering at temperatures above 900 °C. The printed cores showed comparable or better properties to commercial counterparts. 

## 5. Stereolythography

**Figure 18:**
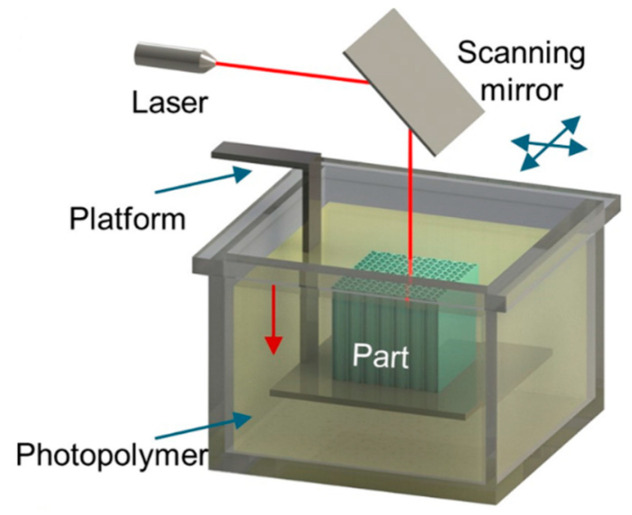
Concept scheme of SLA technology [70].

The SLA method uses a special vat with a liquid photo-sensitive polymer and a UV laser that scans the surface of the liquid polymer along a given trajectory (Figure 18) [70]. As a result of exposure to UV radiation, a solid 2D layer of a given shape is formed, after which the platform is lowered down by the given layer thickness and the laser scans the next layer according to the new section. Thus, the finished product is formed layer by layer. After printing, the product can be subjected to additional post-treatment with UV radiation. Commonly, the polymers used in SLA include various oligomers, monomers, photoinitiators, and special additives. Monomers and oligomers are polymerized and cured by exposure to UV radiation. Photoinitiators are used to initiate polymerization by forming active bonds under the action of UV radiation. Typical materials used in SLA are acrylic and epoxy resins. 

The main variable parameters in this technology are laser power, layer thickness, exposure time at each point, and scanning speed. This method can be used to obtain various products of complex geometry with a good degree of detail and surface quality. Among the disadvantages of this technology are the high cost compared with FDM-based technologies due to the presence of a laser, the more complex design of SLA printers, the lower productivity, the hazards caused by the used polymer materials, and the need to provide additional measures for safe working with this method. There is another type of SLA technology that is called digital light processing (DLP) [70]. This variant differs in that the layer is cured not by scanning the polymer surface with a laser focal spot but by simultaneously illuminating the entire specified section.

To manufacture magnetic materials using this method, magnetic filler powder is introduced into the resin matrix. In [61], NdFeB powder was used; it was introduced into an acrylate-based resin by mixing it in a centrifuge, and the content of the powder in the composite was up to 92 wt.%. However, magnetic powders, often representing either particles of metal alloys or ceramic compounds, are much denser than any polymer, and in a liquid polymer, they can *sink* to the bottom of the printer’s vat. It leads to an uneven distribution of the filler in the composite. In [104], *a special additional nozzle with a programmable pumping system* was used to supply a metal–polymer mixture containing magnetic particles into the surface layer of a liquid polymer immediately before its treatment with UV radiation. This approach makes it possible not only to solve the problem of uneven distribution of magnetic particles but also to obtain *gradient structures* with a given content of particles in each layer, as well as their distribution over the layer. In addition, the applied magnetic field allows for aligning the magnetic particles towards the required direction. A magnetic fluid introduced into the layer through this additional nozzle has its own limit of the magnetic particles content. For example, in [104], the used magnetic fluid contained 72 wt.% of iron particles with a size of 1–20 μm. Since it is introduced into a pure photopolymer, the content of magnetic particles in the composite generally decreases even more, and it should be taken into account when designing products where a certain level of magnetic properties is required.

## 6. Binder Jetting

**Figure 19:**
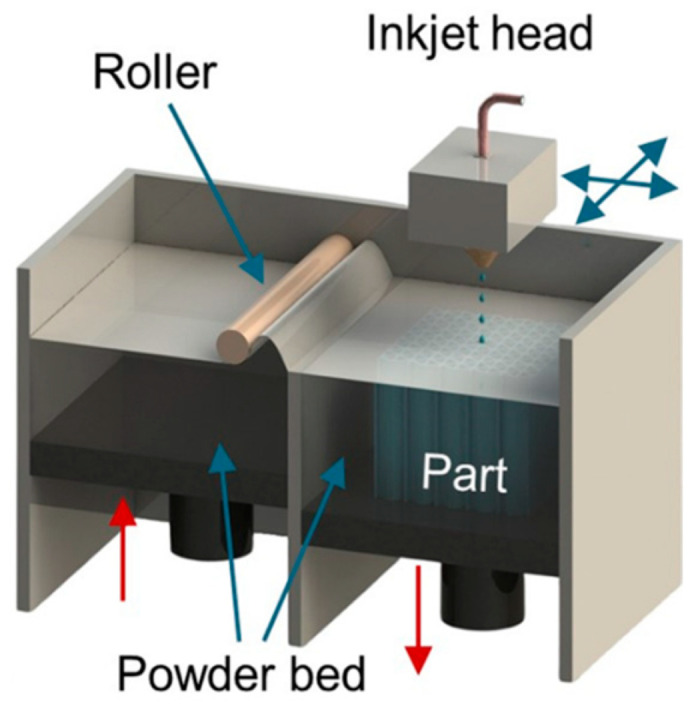
Concept scheme of BJ technology [70].

Another way to fabricate magnetic materials by ATs is to use BJ technology [70,94,126]. In the BJ process, the layer-by-layer selective injection of a binder into a powder located in a special container is performed. The injection is carried out according to a pre-designed 3D-CAD model and a scanning strategy selected. The feedstock material is a fine magnetic powder that is placed in a special tank. A thin layer of powder is applied to the build platform using a special roller and is selectively impregnated by the print head with a quick-drying, low-viscosity polymer binder. For example, an aqueous solution of diethylene glycol (DEG) can be used as a binder [126]. The technology setup is shown in Figure 19. This technology requires some more sophisticated equipment than the previously described technologies but allows for manufacturing a wider range of products.

In the first stage of the BJ technology, a green model is fabricated, which is then dried for curing by evaporating water at a temperature of 100–150 °C for several hours [98], if the binder is water-based, or by cross-linking of molecules during heating of thermosetting polymers [40].

For BJ technology, a spherical powder with good flowability is usually used as a feedstock material. When choosing the optimal particle size, there are competing principles. Thus, coarse powder with a particle size of more than 100 μm provides better flowability, but this increases the minimum thickness of the printed layer. Finer powder with a particle size of 25–100 microns has poorer flowability, but it provides better sintering behavior due to the higher specific surface area of the powder particles and, consequently, the larger contact area between the particles [127]. When using powders with smaller particles, less than 25 µm in size, the powder may adhere to the roller and the quality of the printing process may decrease. However, in some cases, submicron powders with particle sizes up to 0.3 µm are also used [128]. 

In [98], a spherical NdFeB powder with an average particle size of 70 μm was used. The volume fraction of the powder in this work was no more than 46%. When using BJ method, up to 45 vol.% of the magnetic filler can be introduced into a polymer matrix [98]. It is inferior to IM and FDM technologies and requires new approaches to increase the filler content. The main problem in printing with a coarse magnetic powder is the determination of the leveling speed, which must provide an absence of the displacement of the printed elements in the loose powder layer. Larger powders also lead to a rougher surface of the printed products. To increase the mechanical strength as well as to improve the surface quality, BJ-printed magnets can be coated with urethane resin [98].

A green model, printed from magnetic powder by BJ, can be used as a polymer-bonded magnet. There is no significant heating during the BJ process; consequently, *there is no degradation of the structure and magnetic properties of the initial powder in comparison with the printed green model* [98]. However, due to low adhesion of the magnetic powders to the used polymer binders, the mechanical properties of such magnets are not very high. BJ is usually followed by additional operations of debinding and sintering. These operations can be carried out in two stages to obtain an intermediate brown model or in one stage, since the sintering temperature is much higher than the degradation temperature of any polymer. The sintering results in a higher strength of the printed product. In the case of magnetic materials, higher magnetic properties are also achieved compared with bonded magnets, where up to 60 vol.% of the polymer binder is present. However, after debinding and sintering, the magnets are quite porous. To eliminate porosity after BJ, the resulting product is often infiltrated with low-melting alloys, while the required shape of the product is mainly preserved. It helps improve the mechanical and functional properties of the materials.

In [126], magnets based on NdFeB were fabricated by BJ followed by sintering and infiltration by nonmagnetic eutectic Nd_3_Cu_0.25_Co_0.75_ and Pr_3_Cu_0.25_Co_0.75_ alloys. These compositions were selected not only due to their low melting points but also due their ability to significantly increase the internal coercivity of magnets based on NdFeB [129,130,131]. It is generally believed that a thick interlayer of the phase enriched with rare earth elements and depleted in Fe is necessary for weakening the exchange coupling between the grains of the Nd_2_Fe_14_B phase, which leads to an increased coercivity. The introduction of infiltrating alloys can lead to a change in the volume of the product and, consequently, to a change in the ratio of the magnetic and nonmagnetic phases, which generally affects the magnetic properties of the product. Therefore, it is necessary to optimize the content of infiltrating alloys [132]. For more efficient infiltration, an approach is also proposed to apply pressure, leading to the formation of small cracks, along which the infiltrating melt spreads more easily [133].

**Figure 20:**
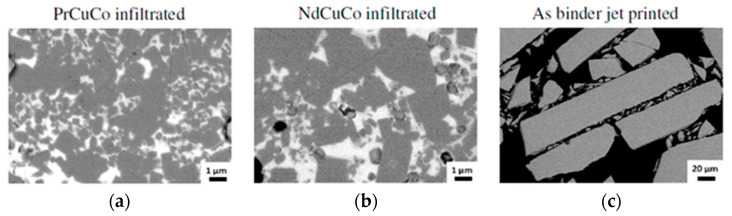
(**a**) Backscattered SEM images of the cross sections of the magnets based on NdFeB in as-printed state after BJ; (**b**) NdCuCo infiltrated; (**c**) PrCuCo infiltrated [126].

In [126], annealing of an uninfiltrated sample at a temperature of 700 °C resulted in a noticeable decrease in its coercivity due to the decomposition of the magnetic phase with the release of soft magnetic α-Fe. After infiltration, remnants of the polymer binder and a finer grain size of the magnetic phase of about 5 μm were still observed (Figure 20), since the infiltration passed most likely along cracks between the particles of the magnetic phase.

When implementing the BJ technology into the fabrication of permanent magnets, *it is also reasonable to apply an external magnetic field in the process of printing to obtain the magnetic anisotropic composite and to increase the value of (BH)_max_*. However, the alignment of the powder particles during the BJ printing process is a significant problem for this technology, since the distance between the print head and the powder layer is very short. Once the powder layer is leveled with the roller, it blocks the print head, which can damage it. In [40], the alignment of powder particles was carried out during the subsequent curing of the printed product in a furnace by placing a sintered NdFeB magnet directly under the product. The measured field of the magnet was about 1 T, which resulted in an increase in (BH)max from 2.4 to 3.8 MGsOe. *Thus, post-treatment with an external magnetic field can become a more preferable approach in the case of using BJ technology for magnetic material manufacturing.*

## 7. Smart Composites

### 7.1. Soft Robots with Magnetic Components

A special area of magnetic polymer–matrix composite application is the *design of smart materials, in particular, various soft robots with a wide range of shapes, sizes, and purposes.* In this case, due to the high flexibility of many polymeric materials along with sufficient strength, they can be easily actuated into various types of movement from torsion and bending to translational movement due to tension and compression. The introduction of magnetic particles, both hard magnetic and soft magnetic ones, into polymers gives functional magnetic properties to the polymer matrices. When there is an external magnetic field, free magnetic particles begin to move along the field lines. Being in the polymer in sufficient quantity, they are able to set the entire composite in motion under the action of the magnetic field. Moreover, the field can be set not only as a constant but also variably with a certain frequency and with a variable direction, for example, rotating [39]. Thus, a controlled external magnetic field is the main driving force of robots based on polymer matrices with magnetic fillers. The advantage of a magnetic field as a motion source is *a very fast response of the actuator system to the external stimulus.* It can be several times faster than the response caused by other sources, such as, temperature effects in the case of actuators exhibiting a shape memory effect. 

The authors of [39,134] provided a rather broad review of such soft robots based on magnetic polymer–matrix composites. Soft robots with a magnetic actuator were divided into two main groups: robots operating under the action of a magnetic force and robots operating under the action of a magnetic torque. The magnetic force usually provides a higher efficiency of movement, and there are less requirements for drive devices. In comparison, the magnetic torque is more attractive in terms of its greater multi-functionality and higher precision in control, especially for the activation of transformer robots and micro robots. It is noteworthy that these two actuation methods are not mutually exclusive, and there are prospects for combining them to create many different methods [39].

**Figure 21:**
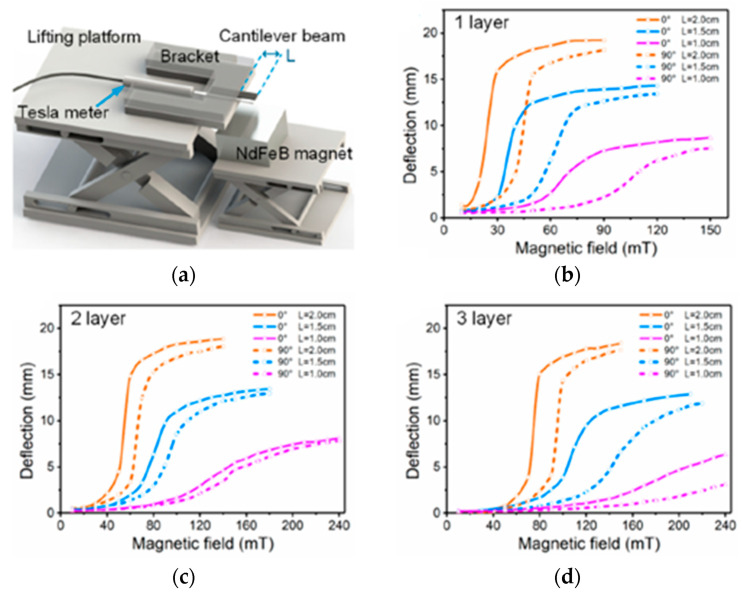
(**a**) Schematic illustration of the cantilever beam test system; (**b**–**d**) the deflection versus the magnetic field curves of the cantilever beam [84].

In magnetic actuation, an important parameter is the response to a magnetic field. In order to study this response, in [84], several cantilevers of different lengths and with different numbers of layers were fabricated by FDM from thermoplastic rubber with 50 wt.% of carbonyl iron (CI). One end of a cantilever was attached to the platform, and the free end was bent under the action of an external magnetic field generated by a permanent magnet and directed along the thickness of the cantilever with a variable field strength. In this case, the dependence of the bending value on the external magnetic field was nonlinear due to the nonlinearity of the field change in the cantilever zone, since the bending resulted in less distance between the cantilever end and the magnet, while the field sensor remained fixed (Figure 21).

As shown in [84], a cantilever printed perpendicular to the filament length showed a greater deformation than a cantilever printed parallel to the filament length due to the lower modulus of elasticity of the former. A lower modulus of elasticity is associated with greater porosity at this printing strategy, as was shown above. In addition, thinner cantilevers with fewer layers have a lower modulus of elasticity and, consequently, a greater tendency to deformation. With the same number of layers, a longer cantilever can generate more strain at the same magnetic field. Thus, to manufacture efficient magnetic actuators, the selection of printing strategy, the shape and dimensions of the active element of the actuator is even more important than the selection of materials with the best properties.

**Figure 22:**
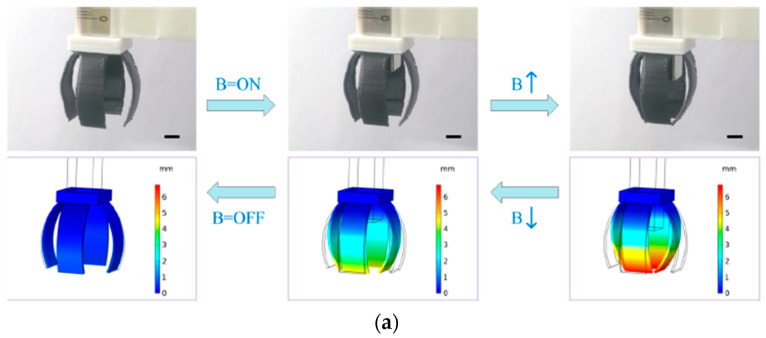

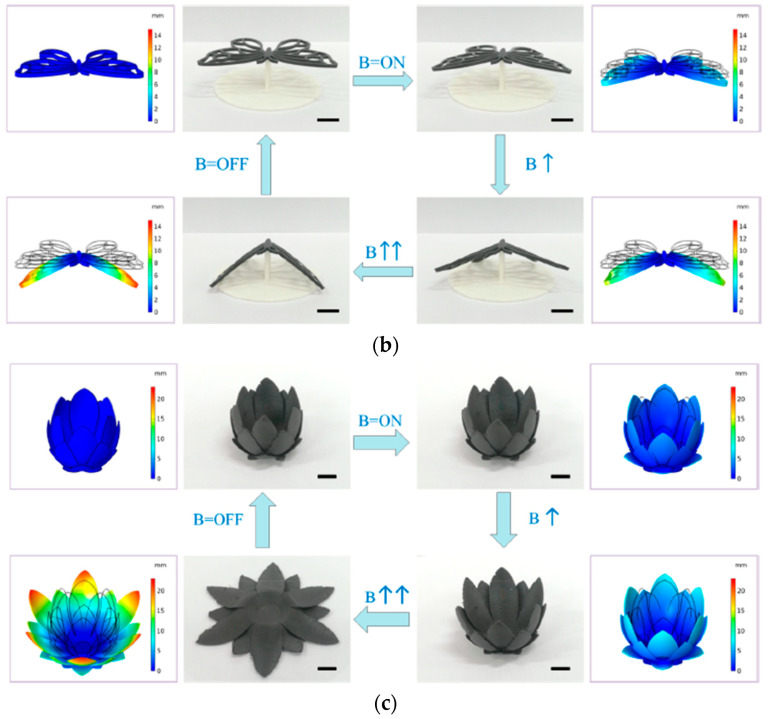
(**a**) Magnetic-field-induced deformation and finite element simulation of the tentacle-like biomimetic magnetic actuator; (**b**) the butterfly like biomimetic magnetic actuator; (**c**) the flower-like biomimetic magnetic actuator [84].

The additive manufacturing of polymer-based magnetic composites is actively utilized for the manufacture of *biomimetic actuators of various shapes and purposes*. In [84], magnetic actuators in the form of octopus tentacles, a butterfly, and a lotus flower were printed by FDM from a rubber filament filled with carbonyl iron. The tentacles, wings, and petals can be effectively manipulated using an external magnetic field, as shown in Figure 22. *Such actuators open up broad prospects for designing various biorobots, including those for medicine*.

**Figure 23:**
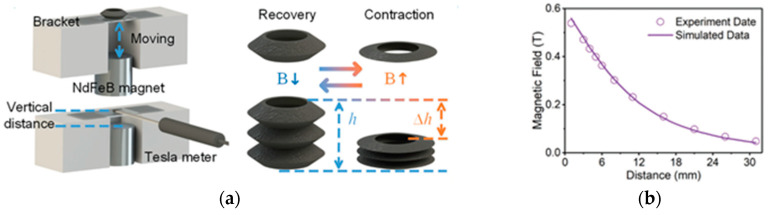

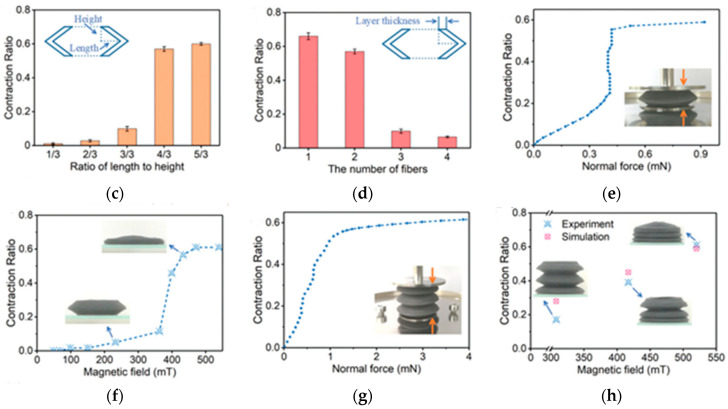
(**a**) The deformation behavior analysis for the hose actuator: The schematic diagrams of the test system; (**b**) the magnetic field versus the distance from the NdFeB magnet; (**c**) effect of the ratio of length to height on the contraction ratio for the hose unit; (**d**) effect of the layer thickness on the contraction ratio for the hose unit; (**e**) the contraction ratio versus the normal force for the hose unit; (**f**) the contraction ratio versus the magnetic field for the hose unit; (**g**) the contraction ratio versus the normal force for the hose actuator; (**h**) the contraction ratio versus the magnetic field for the hose actuator [71].

In [71], a super-flexible hose actuator (Figure 23) was fabricated by screw extrusion. To study the ability of the actuator to be deformed under an action of magnetic field, tests were carried out to estimate its compression at various magnetic fields and geometrical parameters of the actuator. Schemes of typical compressive deformation processes are shown in Figure 23a. The compression ratio can be calculated by measuring the change in actuator height. It was defined as the parameter 𝜑 = ∆h∕h, where h and ∆h are the initial height and the height change, respectively (Figure 23a). When a permanent magnet generating an external magnetic field approaches the actuator, the magnetic field changes non-linearly, similarly to that in [84] (Figure 23b). A larger field causes a larger deformation.

An increase in the length parameter to the height parameter ratio resulted in a larger compression ratio at the same magnetic field (Figure 23c) [71]. The compression ratio increased from 0.01 to 0.6 with a compression ratio change from 1/3 to 3/5. When increasing the number of layers from 1 to 4 (thickness was from 0.8 to 3.2 mm), the compression ratio decreased from 0.66 to 0.07 (Figure 23d). The increased rigidity was associated with a decrease in the degree of folding and an increase in the thickness of the layer, which weakened the effect of the magnetic field on the deformation. To evaluate the compression performance of the actuator, a static load was applied (Figure 23e). The actuator was placed on the rheometer table. The force transducer was used to measure the normal force for various displacements under the load. In the range of small force values, the compression ratio increased linearly with the growth in the normal force. When the normal force reached 0.4 mN, the structure became unstable and lost its bearing capacity, and the compression ratio increased sharply from 0.25 to 0.55. Then, with a further increase in the normal force, the compression ratio did not change due to the densified structure. Figure 23f shows the compression process of a sleeve drive under various magnetic fields. According to the compression experiment, the curves can be divided into three stages. The compression ratio slightly increased in a small magnetic field. When the magnetic field was large enough, the magnetic force acting on the hose actuator increased significantly with an increase in the strain due to the magnetoelastic coupling. The non-linearly increasing strength of the magnetic interaction led to the fact that the structure began to continuously compress until it practically overlapped.

**Figure 24:**
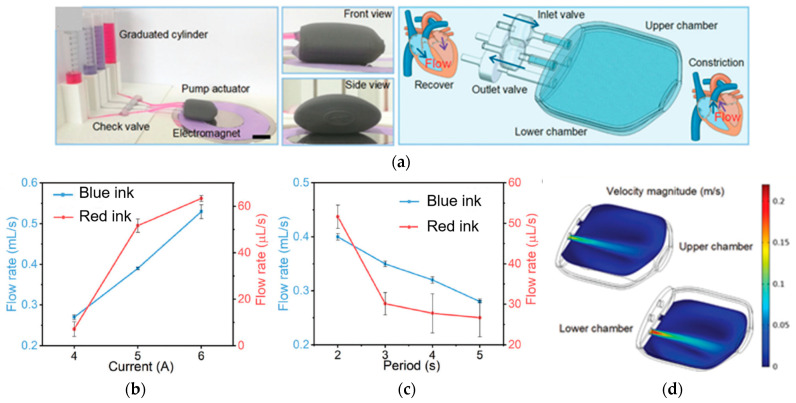
(**a**) Demonstration experiment for the pump actuator: the digital image and schematic diagram of the pump actuator (scale bar: 2 cm); (**b**) plot of flow rate versus current; (**c**) plot of flow rate versus period; (**d**) the simulated results for the fluid velocity of the cross section [71].

Thus, by changing the external magnetic field and using a certain configuration of the actuator, it is possible to realize various maximum deformations and to effectively use this type of actuator in various installations for generating mechanical work. In [71], it was shown that with the use of hose actuator principles, it is possible to print a sucker actuator and a tubular actuator, which can later be converted into a working pump by printing structures with different cross sections. *In particular, it is possible to print pumps that are close in function to the heart muscle* (Figure 24).

To improve the response efficiency, a time-varying magnetic field generated by an electromagnet can be used. When a magnetic field is applied, the top of the pump deforms and contracts under the magnetic force, while the bottom contracts due to the compression between the structure and the electromagnet. In this case, the deformation of the top part is less than that of the bottom part. The asymmetric deformation will generate different pressures at the same time, which causes the inlet check valve to close and the first outlet valve to open, pumping the liquid in it through the check valve.

In [135], it was shown that, *by using polymer–matrix magnetic composites, it is possible to convert mechanical energy into electrical energy by changing the distribution of the magnetic field in the process of deforming such composites*. In particular, the mechanical energy of raindrops can be used to deform a composite product [136]. In [135], the authors developed a flexible magnetic device with a superhydrophobic surface, which makes it possible to provide mechanoelectric conversion due to falling water drops. 

The flexible mechanoelectric conversion device consisted of a magnetic top and an elastic stem-like bottom part with a coil inserted into the base. The top magnetic part was made by FDM from a composite filament based on thermopolyurethane (TPU) with a NdFeB magnetic filler. When printing, the particles were aligned vertically by a pulsed magnetic field. The resulting pimply surface had an increased area, that enhanced the probability of its contact with the falling drops. Next, the surface was treated with a solution containing silicon dioxide particles to improve its hydrophobic properties. The bottom stem-like part was made by jet spraying of a photocurable polymer with UV-treatment of each layer. This method can be used to obtain flexible products with a low elastic modulus. At the following stage, the top part, the bottom part, and a conductive coil were bonded together using an ordinary glue to manufacture the finished device.

To test the device [135], water was periodically dripped onto it from a height of 40 cm in portions of 55 μL. Since the bottom part is made in the form of a rod of an elastic polymer with a low elastic modulus, it bends quite easily under a falling drop. Due to the hydrophobic coating on the top, drops do not linger on it and the device quickly returns to its original shape. With these movements, the distance between the magnetic top and the coil at the bottom changes. In this case, the magnetic flux through the coil also changes, which leads to the generation of electricity from the falling drop. Regular movement of the magnetic part up and down due to the constant action of water drops lead to the generation of an electric current of 12.56 μA with a voltage of 1.6 mV, which is generally higher than that for many existing mechanoelectric transducers based on piezoceramic and tribological effects [135,137,138]. The resulting device can convert the potential energy of water droplets into electricity, being an autonomous sensor without external power. To increase the efficiency of the device, it is possible to use the higher content of the magnetic filler or to increase the thickness of the top part. However, this also increases its mass and, consequently, reduces the amplitude of movement of the device, which requires the search for the optimal combination of various technological and geometric parameters in the development of new similar devices.

**Figure 25:**
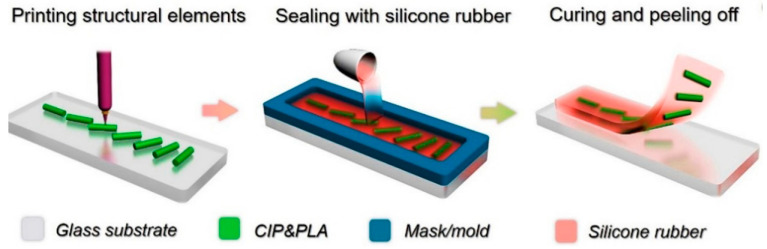
Schematic illustrations of 3D printing and encapsulation with a silicone rubber [139].

**Figure 26:**
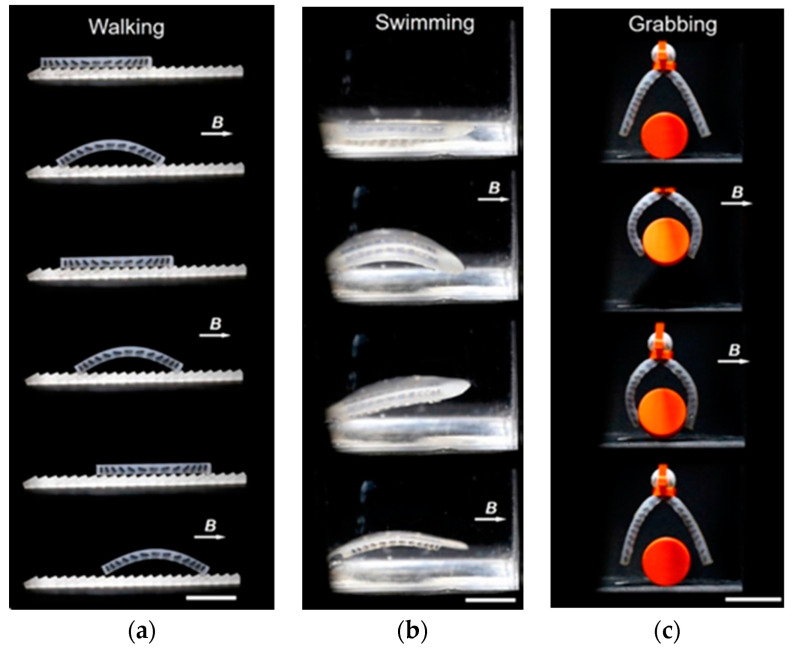
(**a**) Design of soft robots and actuator on the basis of shape-programmable magneto-active soft materials: walking motion of the inchworm-like soft robot on serration plate. The bending of the robot is driven by the uniform magnetic field (UMF), and it is restored by the coupling of gravity and elastic forces. The white scale bar is 10 mm; (**b**) swimming of the manta ray-like soft robot under water. The swing of the swimming robot is also driven by the UMF. The white scale bar is 10 mm; (**c**) grab and release of the soft gripper. The gripping action is driven by the UMF, and the weight of the cylindrical object is 15.3 g. The white scale bar is 20 mm. In these cases, the intensity of the UMF is 300 mT, and the direction is as indicated by the arrow [139].

In [139], the FDM method was used to obtain single tracks of a composite filament based on PLA and carbonyl iron on a glass substrate. Next, a mold was placed onto a glass substrate for pouring liquid silicone rubber that can be cured at room temperature. Thus, the magnetic tracks were completely covered with a soft substance (Figure 25). In this case, the magnetic tracks can be oriented in any way in order to set a required distribution of magnetization depending on the engineering purposes. *Such silicone strips with magnetic tracks can be used as simple actuators or can be utilized for the development of more complex soft robots.* By changing the shape and geometry of the soft robot in [139], the possibility to perform three main functions was obtained: walking, swimming, and grabbing (Figure 26).

In addition to the use of certain ATs for fabricating devices from magnetic polymer–matrix composites, many researchers often *combine several different technologies, including various ATs and conventional technologies that can be used simultaneously*. Thus, in [140], a soft robot was printed from pure ABS plastic using soft lithography methods for soft elements and the FDM method for more rigid elements required to connect the flexible parts. Next, magnetic rings made of NdFeB alloy with inner pneumatic channels were introduced into the printed robot to provide quick remote assembly and disassembly of the robot.

**Figure 27:**
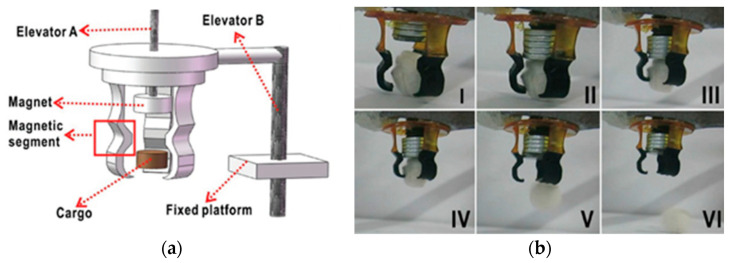
(**a**) Schematic illustration of the magnetic driving gripper set-up; (**b**) demonstration of the 3D-printed gripper achieving the task of grabbing and transporting a cotton ball controlled by a magnet [141].

In [141], the multimaterial DLP technology (Figure 27) was used to print a soft gripper based on a magnetorheological actuating system. Fe_3_O_4_ ferrite was used as a magnetic filler, and the matrix was photocurable resin consisting of acryloyl-modified polyethylene glycol (PEG), 2[(butylcarbamoyl)oxy]ethyl acrylate, and cyclic trimethylpropaneformacrylate (CTFA) as a reaction diluent and 2,4,6-(trimethylbenzoylethoxy, phenylphosphine)oxide as a photoinitiator. The printed grip was actuated by a magnet generating the magnetic field needed to move the fingers (tested with a cotton ball).

**Figure 28:**
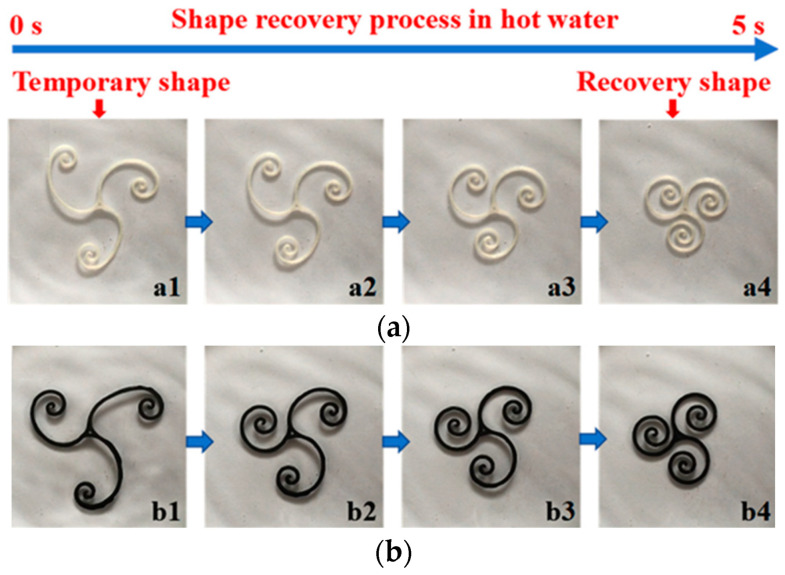
Deployable behaviors of 4D-printed structures in hot water: (**a**) PLA; (**b**) PLA/Fe_3_O_4_ [144].

**Figure 29:**
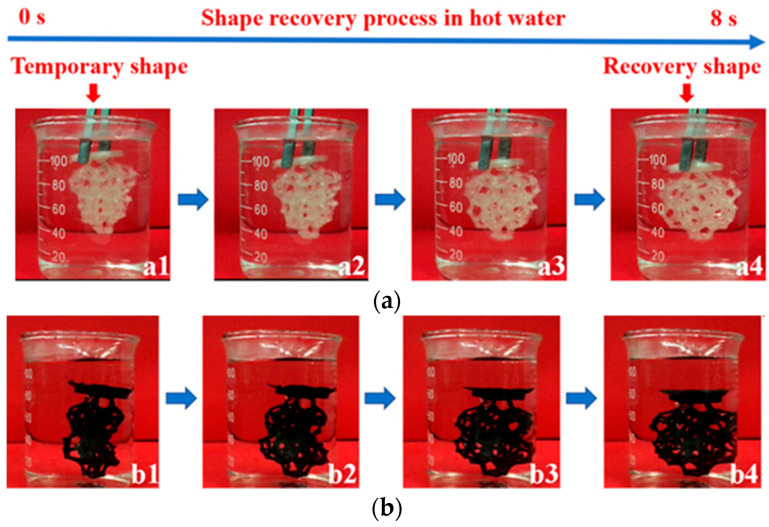
Expansion behaviors of 4D-printed complex structures in hot water: (**a**) PLA; (**b**) PLA/Fe_3_O_4_ [144].

**Figure 30:**
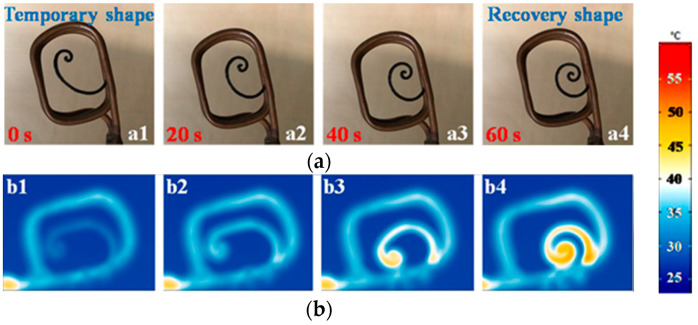
Magnetic-field-triggered shape recovery behavior of 4D-printed structures with 15% Fe_3_O_4_ at 27.5 kHz: (**a**) real process; (**b**) thermal distribution [144].

Soft robots and actuators driven by a magnetic field can be classified as so-called smart devices and smart materials. Additionally, 3D printing technologies for making smart materials and devices are now called 4D technologies. Furthermore, *4D technologies include the 3D printing of materials with a shape memory effect, which, in particular, can be induced not only by temperature but also by a magnetic field* [142,143], and 4D-printed shape memory polymers and their composites are currently being widely studied. Potential applications for 4D-printed smart materials are being explored extensively, especially in the field of targeted drugs. In [144], various 2D and 3D structures (Figure 28, Figure 29 and Figure 30) were printed from filaments based on biocompatible and biodegradable PLA and a PLA/Fe_3_O_4_ composite with a filler content of 15 wt.%.

The shape memory effect of the 2D and 3D structures was studied using hot water [144]. This process included three stages: (1) the printed sample was subjected to a deformation at 80 °C; (2) the deformed sample was cooled down to room temperature to obtain a temporary shape; and (3) the deformed shape returned to its original shape when reheated to 80 °C. Then, 4D-printed PLA and PLA/Fe_3_O_4_ flower-like 2D structures recovered their original shape in hot water within several seconds (Figure 28).

For bone-like 3D structures, the shape memory effect is similar to that of the 2D structures, but the rate of shape recovery was a little lower due to the presence of a higher number of structural details (Figure 29). The coefficients of shape recovery and shape fixation ranged from 95.8% to 96.9%, respectively. *The concept behind using magnetic composites for bone-like structures is that the magnetic nanoparticles in the polymer matrix oscillate in an alternating external magnetic field and generate heat. The generated heat provides energy for shape recovery.* As shown in Figure 30, the shape recovery of the printed 2D composite structure occurs when subjected to an alternating magnetic field at a frequency of 27.5 kHz. The temporary form returned to its original state in 60 s. Figure 27b shows the recovery time and temperature distribution of a 2D structure at 27.5 kHz. The magnetic particles were evenly distributed, causing a uniform temperature change throughout the structure. The surface temperature of the structure was about 40 °C, which is close to human physiological conditions and within the allowable body temperature, making it applicable *in the field of biomedicine, in particular for bone tissue repair engineering*.

**Figure 31:**
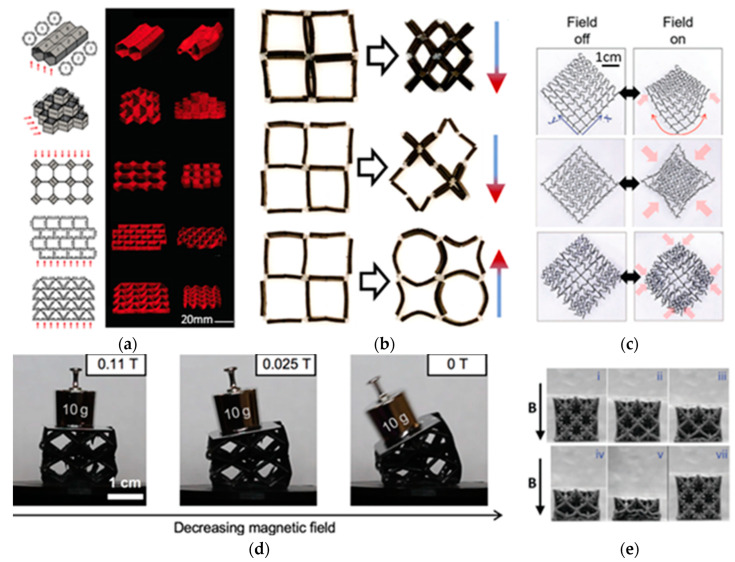
(**a**) An emerging mechanism for active metamaterials is magnetism: a novel DIW approach is used to tune the magnetic pole orientation of different regions during printing that leads to intricate deformations that can be well predicted; (**b**) metamaterials that use an asymmetricfolding joint can achieve drastically different configurations under opposite magnetic fields; (**c**) a DIW-printed metamaterial that floats on water harnesses magnetic and capillary forces to achieve interesting deformations; (**d**) an SLA-printed hollow lattice is filled with a magnetic ink that can be used to adjust the lattice stiffness under a magnetic field; (**e**) a 3D-printed scaffold allows for the injection of a magnetic ink followed by the dissolution of the scaffold. Applying a magnetic field changes the acoustic absorption properties [149].

In [114], the magnetic-field-assisted DIW method was used to print structures that could initiate a change in the shape of printed structures with complex but predictable deformations due to the presence of magnetic particles (Figure 31a). In [145], the same system of materials was used to manufacture metamaterials with asymmetric joints and tunable stiffness and Poisson’s ratio, which can exhibit well-defined deformation modes under opposing magnetic fields, as shown in Figure 31b. In [146], the DIW method was used to achieve magnetization control at the voxel level. This new capability was combined with an evolutionary algorithm to develop the correct magnetic orientation for complex active structures. *This potential of ATs can be used in the future to design and fabricate highly complex magnetically active metamaterials*.

In [147], the DIW method was used to print magnetic active metamaterials consisting of iron particles introduced into polydimethylsiloxane (PDMS). The printed structures were placed onto the water surface and compressed in the presence of a vertical magnetic field due to a combination of magnetic forces and water capillary forces (Figure 31c). In [148], tunable acoustic metamaterials were fabricated using the DIW method. Elastic metastructures provide extended control over the propagation of elastic waves, in particular, due to their ability to exhibit frequency band gaps in which elastic waves cannot propagate. Band gap frequencies are often rigidly determined by materials and the metastructure geometry during the design process. The authors of [148] developed a tunable metamaterial that uses the coupled magneto-mechanical response of magneto-active elastomers to provide active band gap control. It was shown that the band gap of a lattice metastructure can be controlled in a continuous frequency range by applying a magnetic field remotely. The tuning of a band gap depends not only on the strength of the applied magnetic field, but also on the interaction between the magnetic field and the geometry of the metastructure. Thus, the combined effects of geometry and the increase in rigidity due to the magnetic field are new design parameters for the customization of metastructures, allowing for the design and fabrication of new smart structures that have the ability to control their own vibrations flexibly.

In [150,151], the SLA and DLP technologies were used to fabricate hollow lattice structures filled with magnetic ink, which fill the cells and give them customizable mechanical properties in a magnetic field (Figure 31d,e). These complex structures can exhibit widely tunable acoustic properties with a remote magnetic field. In [152], the selective action of UV radiation was used to encode hard magnetic particles in flat materials with an arbitrary three-dimensional orientation on a micro-scale. Three-dimensional magnetization profiles were also achieved by rotating the magnet during photocuring, allowing for higher-order complex bending and twisting for microrobot motion. In [153], the rotation of the magnetic field during SLA printing was also used to control the orientation of local magnetic dipoles.

In [154], the process of electron beam lithography was used to deposit nanomagnets onto a soft/hard silicone membrane. The hard parts were connected by softer elements that acted as hinges. In this work, several types of magnetic particles with unique hysteresis loops were used, which makes it possible to program different particle directions independently. *This variability allows for fabricating interesting shapes on a small scale*.

### 7.2. Smart Fillers

One of the interesting directions in the fabrication of smart polymer-based materials is *the use of smart magnetic materials as fillers, such as magnetostrictive alloys and alloys with a magnetically induced shape memory effect (MISME)* [155]. Their feature consists of changes in the linear dimensions of the product under the action of an external magnetic field. For magnetostrictive materials, the relative change in size is up to 0.2%, and for materials with MISME, it can reach up to 12%. 

Magnetostriction is a characteristic of all magnetic materials, though its values can dramatically differ. Terfenol-D has the largest magnetostrictive effect and magneto-mechanical coupling coefficient at room temperature. However, its brittleness and high eddy current losses at high frequencies hinder its practical application. The method of combining Terfenol-D particles with polymers, proposed in [156,157], proved to be an effective way to overcome these drawbacks. Although the magnetostrictive coefficient of the composite is less than that of a monolithic Terfenol-D, it has lower eddy current losses at higher operating frequencies. In composite form, the material is less brittle and easier to manufacture than a monolithic Terfenol-D.

MISME is exhibited due to the occurrence of thermoelastic martensitic transformation, the temperature of which can vary due to the action of a magnetic field of different intensities and is best studied for Heusler Ni-Mn-Ga alloys [158]. The occurrence of giant reversible magnetic deformation in a magnetic field observed in these alloys is due to the rearrangement of twin martensitic variants under the action of an external magnetic field due to the strong magnetocrystalline anisotropy and high mobility of twin boundaries. Similar properties are also observed in alloys such as Ni_2_MnAl, Co_2_MnGa, Co_2_MnAl [159], Fe-Pd [160], Fe-Pt [161], Co-Ni-Ga [162], Co-Ni-Al [163], and some others. 

**Figure 32:**
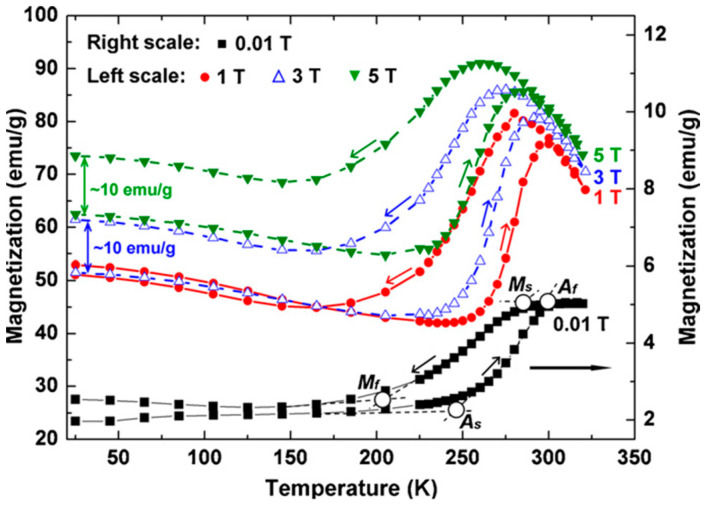
Thermo-magnetization curves of the composite measured in magnetic-field strengths of H = 0.01, 1, 3, and 5 T. For clarity, different scales of magnetization are used. Arrows near the curves indicate the different heating or cooling routines [164].

In [164], a polymer-based composite with a 50 vol.% of Ni_45_Co_5_Mn_36.6_In_13.4_ particles was studied. Figure 32 shows the thermomagnetic curves of the composite at a magnetic induction up to 5 T. At 0.01 T, austenite started to transform into martensite at 284 K and ended at ∼200 K. The reverse transformation started at ~250 K and ended at 299 K. With an increase in the external magnetic field, the temperatures of the martensitic phase transformation decreased. This trend indicated that the polymer-based composite with a Ni_45_Co_5_Mn_36.6_In_13.4_ filler has the potential for magnetically induced martensitic transformation.

The phase transformation interval for the composite is about 50–80 K, which is much wider than that of the original alloy (~10 K). The authors of [164] attributed this to higher internal stresses in the powder in comparison with the initial ingot, which were made by mechanical milling. These stresses were partially preserved even after annealing. Additional stresses can also be induced by the polymer matrix. The achieved high values of deformation caused by the magnetic field, together with good ductility and low cost, make polymer–matrix composites based on a Ni–Co–Mn–In filler a potential material for the manufacture of magnetic actuators.

The composites mentioned above were prepared by compacting a mixture of a polymer with magnetic particles to obtain simple cylindrical specimens. In this case, quite interesting results were obtained. However, at this moment, there are no published studies devoted to using ATs for manufacturing polymer-based composites with smart magnetic fillers. *The use of polymer additive technologies can bring the fabrication of smart materials to a new level and seems to be rather promising*.

## 8. Conclusions

The present review showed the applicability of polymer additive technologies such as FDM, DIW, SLA, BJ to production of magnetic materials when introducing magnetic particles into a polymer base. The most researched compositions of magnetic fillers in these field are rather well-known hard magnetic materials (Nd-Fe-B, Sm-Co, Alnico and hard magnetic ferrites such as BaFe_12_O_19_) and soft magnetic materials (Fe, special steels and soft magnetic ferrites such as F_3_O_4_, NiZn, NiFe_2_O_4_), though there are also works devoted to search of new compositions such as Ce- Fe_16_N_12_ based magnets and a mixture of several compositions such as SrFe_12_O_19_ + NdFeB aimed in improving their performance.

Since the heat sources used in these technologies for melting or curing the polymer matrix have rather low power, they do not significantly change the structure of the magnetic fillers and their intrinsic magnetic properties, such as H_ci_. Thus, one of the main factors in the formation of magnetic characteristics of polymer-based composites is the filler content, as B_r_ and B_s_ are proportional to the volume fraction and (BH)_max_ are proportional to the square volume fraction. However, too high of an increase in the volume fraction leads to some technological difficulties, and each technology has its own limitations.

Thus, FDM technology allows for obtaining a filler content up to 80 wt.%. At the same time, DIW technology provides a content up to 98 wt.% due to the lower viscosity of the used polymers. DIW technology also provides lower porosity and better product detail and surface quality than FDM. Nevertheless, FDM technology is one of the most wide-spread and affordable polymer 3D printing methods due to it having some important advantages, such as the relative simplicity of FDM printers. This technology does not result in blurring tracks, and it is more productive.

The SLA method can be used to obtain various products of complex geometries with a good degree of detail and surface quality. Among the disadvantages of this technology are the high cost due to the presence of a laser, the complex design of SLA printers, the rather low productivity, and the hazards caused by the used polymer materials. It is also difficult to obtain a uniform distribution of magnetic particles in the liquid polymer. At the same time, there are studies proposing a method to overcome this issue by using an additional nozzle to supply a metal–polymer mixture containing magnetic particles into the surface layer of a liquid polymer immediately before its treatment with UV radiation.

BJ technology requires some more sophisticated equipment and more restricted requirements regarding particle size and shape but allows for manufacturing a wider range of products.

Since it is impossible to achieve a filler content of 100% when using polymer Ats, the magnetic properties of polymer–matrix composites are always inferior to the properties of sintered magnets of the same composition. However, this review indicates that the described ATs allowed for achieving properties that are comparable, or in some cases superior, to that of bonded magnets produced by conventional IM. To increase the filler content in a polymer matrix, researchers have proposed new approaches, for example, the use of powders with bimodal size distribution.

It was shown that one of the effective ways to improve the magnetic properties of the printed composites is to apply an external magnetic field in the process of printing or post-processing that can be implied to a 3D printer quite easily. It shows better results when using irregular-shape particles, though they increase the viscosity of the composite in comparison with spherical ones. Thus, 3D printing of magnetic polymer–matrix composites is a nontrivial task and demands to find a balance between numerous technological parameters that often compete with each other. 

Despite the lower characteristics of metal–polymer composite in comparison with pure metal magnetic materials produced by metal additive technologies, polymer ATs are often more accessible and easier to implement. They also provide more flexible variation in the product geometry and are already used in the manufacturing of products such as permanent magnets with various shapes and with different surface topologies, transformer cores, shimming elements, and sensors. For the production of such parts, a rather important step is simulation-based design that includes solving direct and inverse problems of magnetization and stray fields by means of FEM.

Polymer ATs are also relatively easy to combine with each other, which makes it possible to fabricate complex structures with various functional elements. One of the most promising areas for polymer ATs is the fabrication of soft robots and actuators that respond to an external magnetic field. Metal–polymer composites with magnetic fillers respond very quickly to an external magnetic field, which generally provides more precise manipulations and can be utilized for the fabrication of different biomimetic actuators of various shapes and purposes, biorobots, heart-like pumps, and shape memory bone-like structures that are promising issues for medicine. But, soft robots can also be used in other technical fields where there is a need, such as in mechanical work and even electricity generation. Magnetic metal–polymer composites produced by Ats are also promising in the design of new highly complex magnetically active metamaterials and metastructures.

Another interesting, but still practically unexplored, direction is the fabrication of magnetic composites with a smart filler that exhibits a magnetically induced shape memory effect. Such composites can promote the development of targeted and customized medicine and smart technologies.

At the present moment, one of the main problems of ATs is its rather low productivity, as well as the limited size of the resulting products that usually do not exceed several centimeters in every dimension. However, active work is underway to solve these problems, and the emergence of new technologies such as BAAM indicates significant progress in this direction. In addition, the already existing 3D technologies remain indispensable in the low-scale production of unique products of complex structures, including miniature products with a high degree of detail.

## Figures and Tables

**Table 1:** Magnetic properties of NdFeB magnets fabricated by different technologies.

Technology	B_r_, mT	H_ci_, kA/m	(BH)_max_, MGOe	Reference
FDM	310–790	688–789	5.3–12.2	[40,41,61,86,94,95]
DIW	300–466	756–954	4.72	[78,94,96,97]
SLA	388	738	-	[61]
BJ	300–420	716–1129	3.8	[40,94,98]
SLS	330–436	522–696	2.1	[61,99]
SLM	700	440	8.6	[100]
Sintering	1005–1140	950–2800	28–56	[94,101]
IM	480–540	636–720	4.6	[40,94]

## Data Availability

No new data was created.
